# High-throughput metabolomics reveals dysregulation of hydrophobic metabolomes in cancer cell lines by *Eleusine indica*

**DOI:** 10.1038/s41598-022-13575-6

**Published:** 2022-06-06

**Authors:** Perng Yang Puah, Dexter Jiunn Herng Lee, Soo Huan Puah, Nik Amin Sahid Nik Lah, Yee Soon Ling, Siat Yee Fong

**Affiliations:** 1 Department of Biomedical Sciences, Faculty of Medicine and Health Sciences, Universiti Malaysia Sabah, Jalan UMS, 88400 Kota Kinabalu, Sabah Malaysia; 2 Biotechnology Research Institute, Universiti Malaysia Sabah, Jalan UMS, 88400 Kota Kinabalu, Sabah Malaysia; 3 Medical Department, Sarawak General Hospital, Jalan Hospital, 93586 Kuching, Sarawak Malaysia; 4 Medical Department, Hospital Seberang Jaya, Jalan Tun Hussein Onn, Seberang Jaya, 13700 Permatang Pauh, Penang Malaysia; 5 Department of Surgery, Faculty of Medicine and Health Sciences, Universiti Malaysia Sabah, Jalan UMS, 88400 Kota Kinabalu, Sabah Malaysia; 6 CAIQ Certification Sdn Bhd Kota Kinabalu, Sabah, Malaysia; 7 Borneo Medical and Health Research Centre, Faculty of Medicine and Health Sciences, Universiti Malaysia Sabah, Jalan UMS, 88400 Kota Kinabalu, Sabah Malaysia

**Keywords:** Metabolomics, Cancer

## Abstract

*Eleusine indica*, which is used in traditional medicine, exhibits antiproliferative activity against several cancer cell lines. However, metabolomic studies to evaluate the metabolite changes induced by *E. indica* in cancer cells are still lacking. The present study investigated the anticancer effects of a root fraction of *E. indica* (R-S5-C1-H1) on H1299, MCF-7, and SK-HEP-1 cell lines and analyzed metabolic changes in the treated cancer cells using ultra-high-performance liquid chromatography high-resolution mass spectrometry (UHPLC-HRMS). Cell metabolic activity assays demonstrated that the cell viability of the three cancer cell lines was significantly reduced following treatment with R-S5-C1-H1, with half-maximal inhibitory concentrations values of 12.95 µg/mL, 15.99 µg/mL, and 13.69 µg/mL at 72 h, respectively. Microscopy analysis using Hoechst 33342 and Annexin V fluorescent dyes revealed that cells treated with R-S5-C1-H1 underwent apoptotic cell death, while chemometric analysis suggested that apoptosis was triggered 48 h after treatment with R-S5-C1-H1. Deconvoluted cellular metabolomics revealed that hydrophobic metabolites were significantly altered, including triacylglycerols, phosphatidylcholine, phosphatidylethanolamine, sphingomyelin, and ceramide, suggesting that apoptosis induction by R-S5-C1-H1 potentially occurred through modulation of phospholipid synthesis and sphingolipid metabolism. These metabolomic profiling results provide new insights into the anticancer mechanisms of *E. indica* and facilitate the overall understanding of molecular events following therapeutic interventions.

## Introduction

In the continuing battle of humans against cancer, which dates as far back as 3000 BC, the earliest written account of cancer is the description of breast cancer found in the Edwin Smith Papyrus^1^. Currently, cancer remains one of the leading causes of premature death among people aged between 30 and 69 years worldwide^2,3^. It is estimated that there were 19.3 million new cases in 2020^2^ and almost 10.0 million deaths from cancer. Furthermore, the incidence of all cancers in Malaysia has been projected to almost double by 2040, from 48,639 to 86,666 new cases^4^. Despite recent therapeutic advancements^5^, this disease continues to affect the quality of life of patients due to the limitations of current cancer treatments.

According to the World Health Organization, there are still many people who rely on herbal medicine as their primary healthcare resource^6^. Therefore, plants have an important role to play in the healthcare revolution. In traditional Chinese medicine, herbs have been used to prevent and treat a wide range of diseases^7^. For instance, the authorities in China encouraged the combined use of traditional Chinese medicine and Western medicine to protect and treat patients infected with the highly infectious novel coronavirus (SARS-CoV2), an approach that has proven to be effective to date^8^. In plants, secondary metabolites are distinct from the components of primary metabolism as they are not involved in regulatory metabolism; rather, they function to protect the plant against predation and microorganism invasion^9^. Decades of phytochemical research on over 150,000 plant species have revealed that secondary metabolites including phenolics, flavonoids, terpenoids, alkaloids, and sulfur-containing compounds, exert a variety of interesting biological effects^10^. Since 1981, nearly three-quarters (64.9 %) of prescribed anticancer drugs have been plant-derived^11^. Thus, medicinal plants are regarded as important and reliable sources for the discovery of anticancer drugs^12–14^.

Metabolomics is the comprehensive study of a complete set of small metabolites (<1500 Da) within a biological sample^15^. This technique broadens the systems biology view of organisms by bridging the gap between genotype and phenotype^16,17^. Metabolomics is widely applied in studies on plants, animals, medicine, and food. Among the technologies applied in metabolomics, including nuclear magnetic resonance (NMR), gas chromatography (GC), and liquid chromatography (LC) coupled with mass spectrometry (MS), LC-MS is the technique of choice for detecting and determining the elemental composition and molecular formula of an analyte of interest, as it can be performed without a derivatization step. Furthermore, the soft ionization and non-extensive heat application in LC for chromatographic separation reduce the chance of compound degradation during profiling^18^. Endogenous and exogenous metabolic characterizations of therapeutic agents using high-throughput metabolomics allow researchers to evaluate the efficacy and safety of these agents^19^. In cancer research, metabolomic studies have provided comprehensive information and improved the understanding of the underlying mechanisms of cancer pathogenesis and drug effects through the assessment of metabolic changes in cancer cells^20,21^. Thus, identification and quantification of extracted metabolites enable the monitoring of responses to external stimuli in test samples^22,23^.

*Eleusine indica*, also known as wiregrass or goosegrass, is one of the six diploid species of the genus *Eleusine* belonging to the family Poaceae^24,25^. It is commonly found in tropical and sub-tropical regions^26,27^ and is known to possess depurative, febrifugal, diuretic, and laxative properties^25,28,29^. Traditionally, the plant has been frequently used to treat hypertension, influenza, oliguria, and urine retention^25,30,31^, while decoctions of the whole plant are commonly used as antihelminthics and febrifuges^32^. The seeds are sometimes used as a famine food, as well as for the treatment of liver complaints^26^. Extracts of *E. indica* have been reported to exert antiproliferative effects against several types of human cancer cells including Rhabdomyosarcoma cells^33^, lung cancer cells (A549), and cervical cancer cells (HeLa)^34^. Although previous research has revealed the anticancer potential of *E. indica*, there remains a lack of scientific evidence regarding the effectiveness of the plant extracts against other human cancer cell lines, as well as metabolomic studies evaluating the metabolite changes in cancer cells induced by *E. indica*.

Therefore, this study aimed to i) evaluate the effects of *E. indica* root fractions on cell viability and cellular morphology in human non-small cell lung carcinoma (H1299), breast adenocarcinoma (MCF-7), and liver adenocarcinoma (SK-HEP-1) cells; and ii) examine the influence of the most active fraction on the metabolomic profiles of these cells using an LC-MS-based approach.

## Results

**Figure 1:**
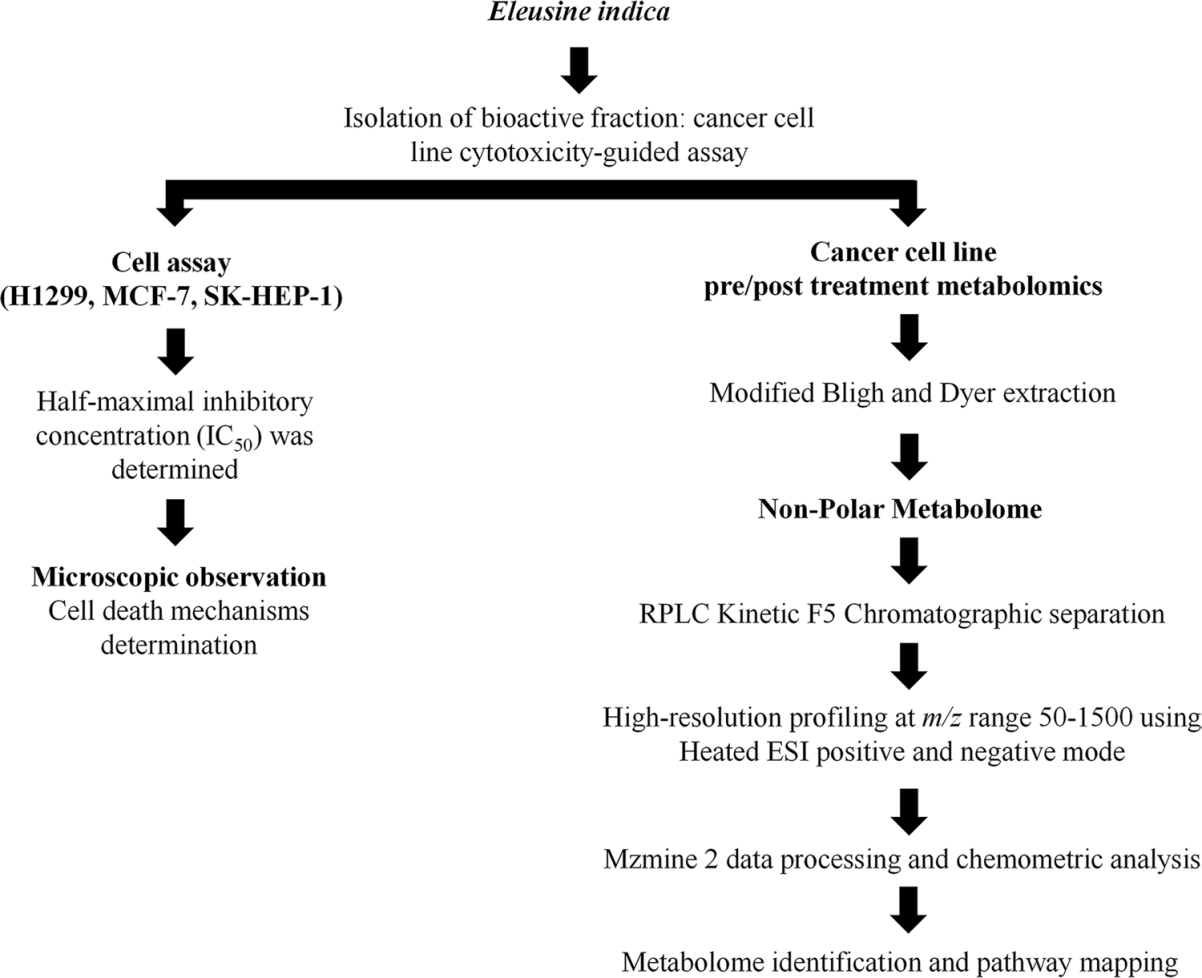
Overall experimental workflow.

The experimental flowchart is illustrated in Fig. 1. Briefly, *E. Indica* was harvested and prepared for the extraction of compounds to be tested against different cancer cell lines. Half-maximal inhibitory concentrations (IC_50_) of the fractions were determined for each cell line using cell viability assays conducted 24, 48 and 72 h after treatment. Subsequently, staining with Hoechst 33342 and Annexin V staining was performed to determine the mechanism of cell death. Concurrently, cell metabolomics was carried out in order to elucidate the cell death-triggering mechanisms; in brief, snap-frozen cells underwent a Bligh and Dyer extraction protocol with modifications to separate polar and non-polar metabolomes. Following this, chromatographic separation of the extracted metabolomes was carried out using pentafluorophenyl columns. Metabolome profiling was carried out at an *m/z* range of 50–1500 using heated electrospray ionization, in both positive and negative modes. Acquired data were pre-processed using MZmine 2^35^, followed by chemometric analysis. Each statistically significant metabolome underwent further compound matching and analysis.

### *E. indica* root fraction R-S5-C1-H1 reduced the number of viable H1299, MCF-7, and SK-HEP-1 cells

**Figure 2:**
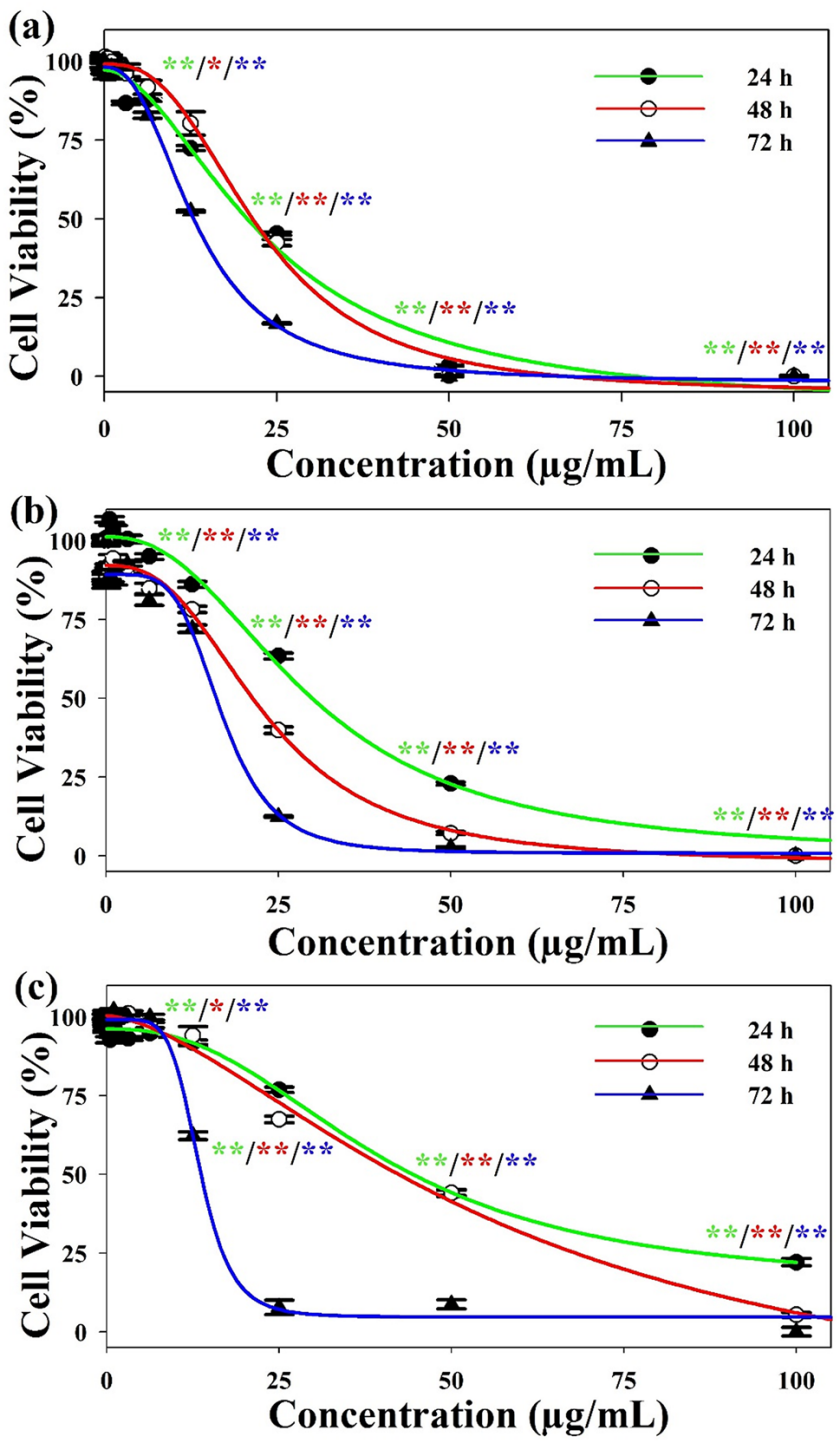
Viability of H1299 (**a**), MCF-7 (**b**), and SK-HEP-1 (**c**) cells following treatment with varying concentrations (0–100 µg/mL) of fraction R-S5-C1-H1 for 24 h, 48 h, and 72 h. Values are expressed as mean ± SEM of three independent experiments; differences were considered significant at p ≤ 0.05 (*) and p ≤ 0.001 (**).

Bioassay-guided screening of *E. indica* extract fractions against H1299, MCF-7, and SK-HEP-1 cells using colorimetric 3-(4,5-Dimethylthiazol-2-yl)-2,5-diphenyltetrazolium bromide (MTT) assays showed that the root fraction R-S5-C1-H1 exhibited the highest level of activity against all cell lines (Figure S1); this fraction was therefore selected for further analysis. As shown in Fig. 2, treatment with increasing concentrations of R-S5-C1-H1 resulted in significantly reduced cell viability in all three cancer cell lines after 24 h, 48 h, and 72 h, with the most marked effects observed at a concentration of 25 µg/mL. For H1299 and MCF-7 cell lines, numbers of viable cells decreased gradually with treatment time, while SK-HEP-1 cells showed a sharp decrease in viability at 72 h. Table 1 shows the IC_50_ values for H1299, MCF-7, and SK-HEP-1 cells after treating for 24 h, 48 h, and 72 h with fraction R-S5-C1-H1; doxorubicin (Dox) was used as a positive control. The highest level of R-S5-C1-H1 activity after 24 h was observed in H1299 cells (IC_50_ = 20.73 µg/mL), followed by MCF-7 (IC_50_ = 29.89 µg/mL) and SK-HEP-1 (IC_50_ = 44.36 µg/mL) cells. Prolonging the treatment up to 72 h resulted in an approximately twofold decrease in IC_50_ values for H1299 and MCF-7 cell lines, and a more than threefold decrease for SK-HEP-1 cells.

**Table 1:** IC_50_ values for fraction R-S5-C1-H1 and doxorubicin in H1299, MCF-7, and SK-HEP-1 cell lines after 24 h, 48 h, and 72 h treatment.

Cell line	IC_50_ (μg/mL)					
	24 h		48 h		72 h	
	R-S5-C1-H1	Dox	R-S5-C1-H1	Dox	R-S5-C1-H1	Dox
H1299	20.73	24.48	21.49	3.70	12.95	2.70
MCF-7	29.89	67.36	21.26	3.58	15.99	1.37
SK-HEP-1	44.36	52.32	42.31	1.87	13.69	1.41

### R-S5-C1-H1 affected cellular and nuclear morphology in H1299, MCF-7, and SK-HEP-1 cells

**Figure 3:**
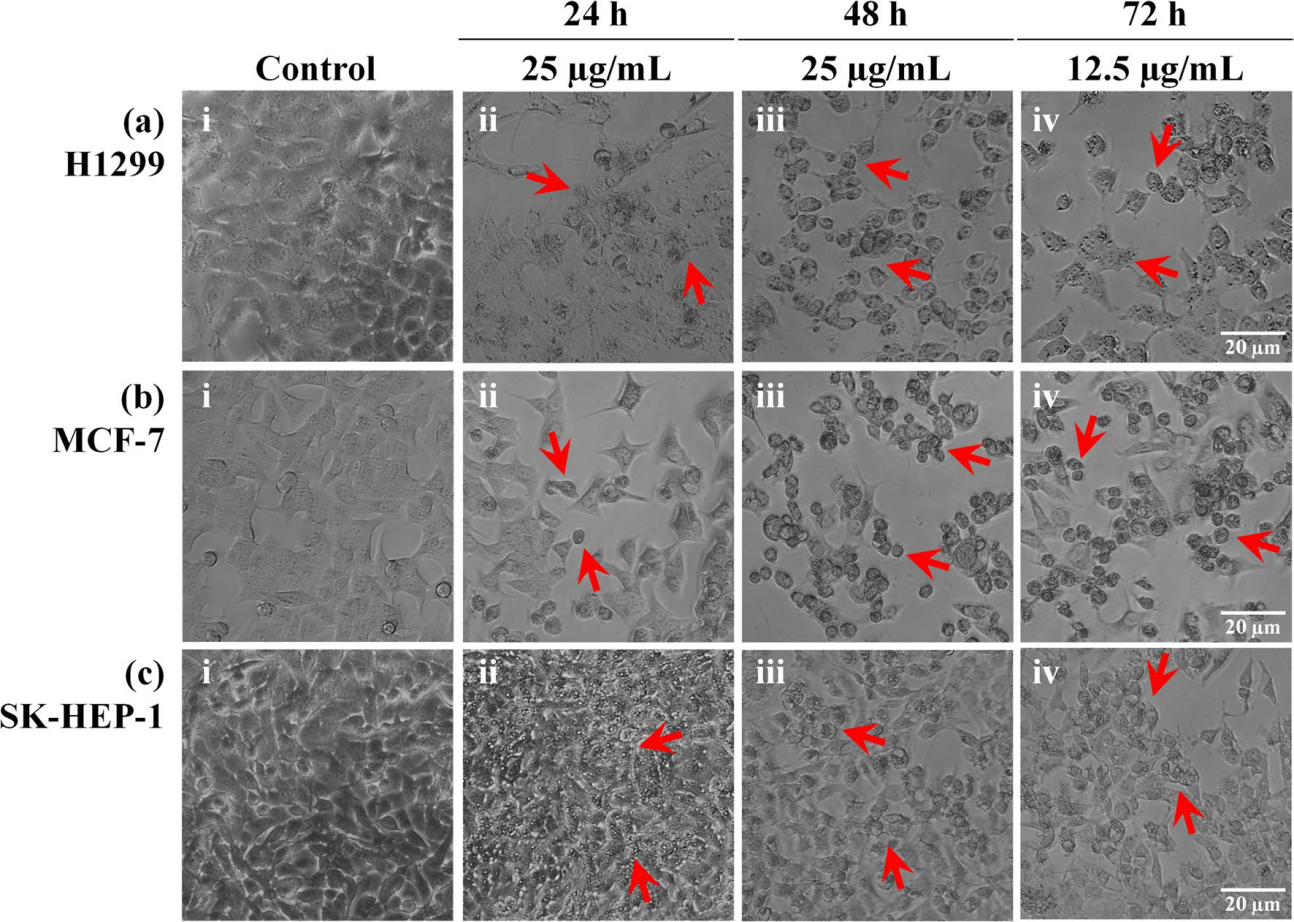
Light microscopy observation of morphological changes, indicated by red arrows, in H1299 (**a**), MCF-7 (**b**), and SK-HEP-1 (**c**) cells treated with 12.5 µg/mL (iv) and 25 µg/mL (ii and iii) of R-S5-C1-H1 for 24 h (ii), 48 h (iii), and 72 h (iv). Untreated control cells appeared adherent with smooth surfaces (i).

When observed under a light microscope, H1299, MCF-7, and SK-HEP-1 cells treated with R-S5-C1-H1 displayed notable cell shrinkage and loss of adherence, while the cells were also observed to be rounded, with irregular and rough surfaces (Fig. 3a–c ii–iv). In contrast, untreated controls for all three cell lines were adherent and exhibited smooth surfaces (Fig. 3a–c i). More noticeable morphological changes were observed when the cells were exposed to longer treatment, in agreement with the results of the cell viability assay.

**Figure 4:**
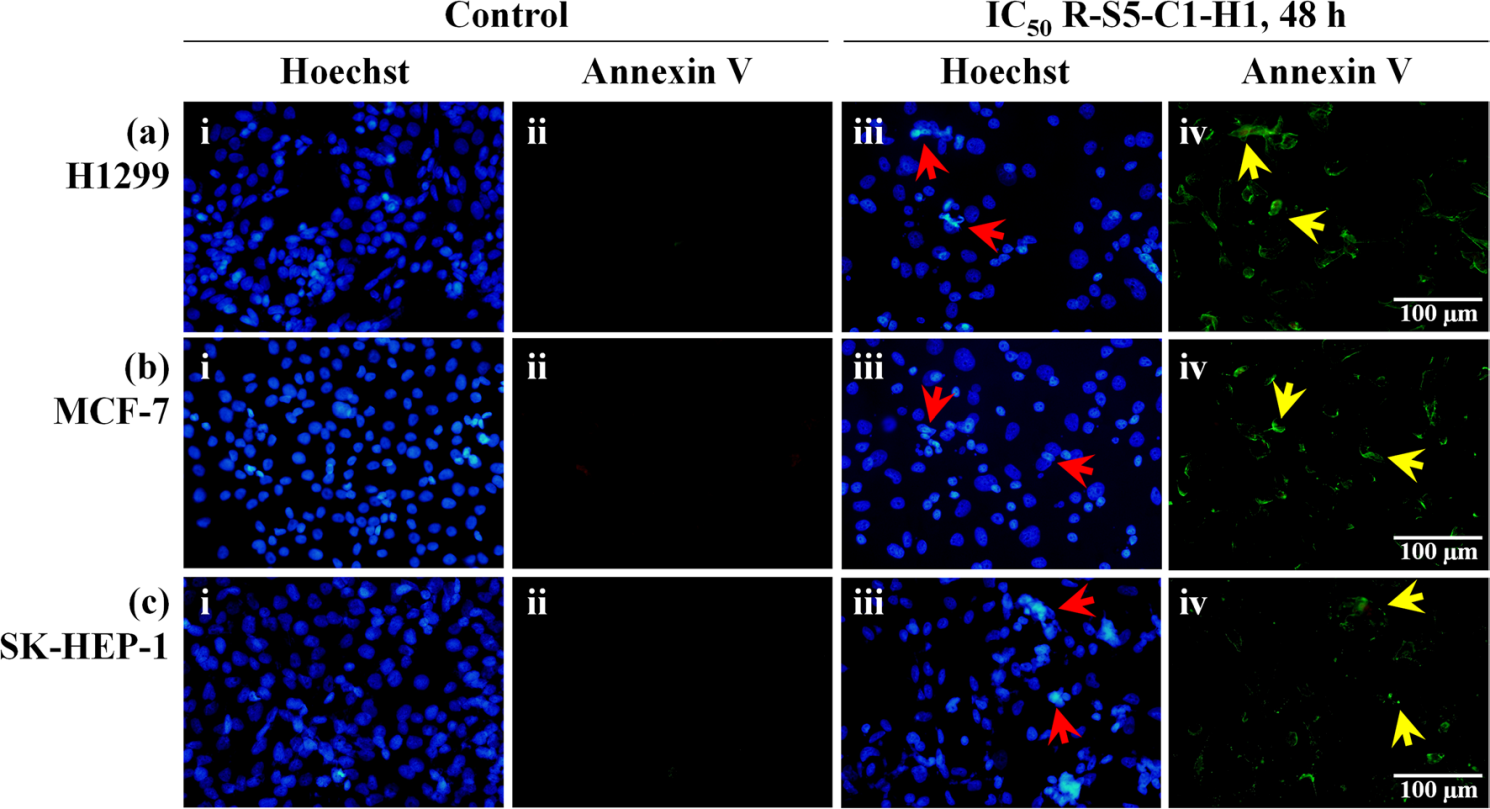
Morphological changes in apoptotic H1299 (**a**), MCF-7 (**b**), and SK-HEP-1 (**c**) cells following treatment with IC_50_ of R-S5-C1-H1 for 48 h, observed by fluorescence microscopy. Hoechst 33342 staining (blue) and Annexin V staining (green) were used to detect apoptotic cells in untreated control (i and ii) and treatment (iii and iv) groups. Red arrows indicate nuclear condensation (bright blue), while yellow arrows indicate phosphatidylserine externalization.

To examine the ability of R-S5-C1-H1 to induce apoptotic cell death in H1299, MCF-7, and SK-HEP-1 cells, Hoechst 33342 and Annexin V fluorescent dyes were used. Hoechst 33342, a DNA-binding dye, was used to detect nuclear condensation and fragmentation, which are major characteristics of apoptosis. Meanwhile, Annexin V, a 36-kDa calcium-binding protein, binds to phosphatidylserine (PS), which becomes translocated from the inner plasma membrane to the external cell surface during apoptosis. As shown in Fig. 4, apoptosis was observed in all three cancer cell lines following treatment with the IC_50_ of R-S5-C1-H1 for 48 h. Morphological changes, namely nuclear condensation and PS externalization, were revealed by Hoechst 33342 staining (blue) and Annexin V staining (green), respectively. The treated cells exhibited bright blue apoptotic nuclei (Fig. 4a–c iii) and green fluorescence (Fig. 4a–c iv), whereas, in the untreated control group, most cell nuclei showed weak homogeneous blue staining (Fig. 4a–c i) and absence of green fluorescence (Fig. 4a–c ii).

### Non-polar metabolome changes induced by R-S5-C1-H1 in H1299, MCF-7, and SK-HEP-1 cells revealed by chemometric analysis

**Figure 5:**
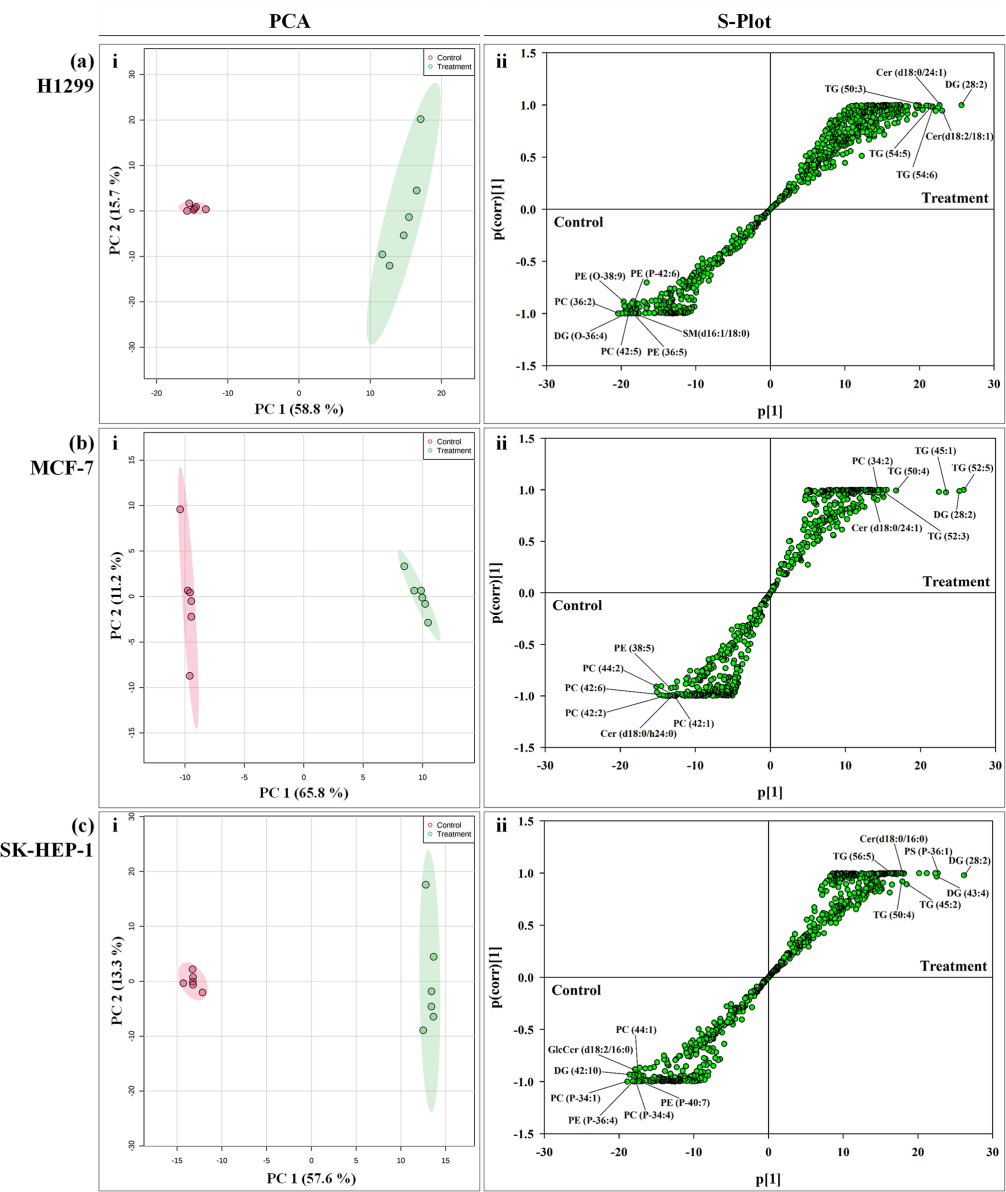
Principal component analysis (PCA) (i) and S-plots (ii) derived from the control and treated non-polar metabolomes of H1299 (**a**), MCF-7 (**b**), and SK-HEP-1 (**c**) cells analyzed in positive ionization mode. Eclipses show the mean ± SD of the sample replicates. For the PCA, red dots represent the control group’s metabolites, while green dots represent the treatment (IC_50_ R-S5-C1-H1, 48 h) group’s metabolites. The labeled metabolites are the significantly perturbed metabolomes in the S-plots.

**Figure 6:**
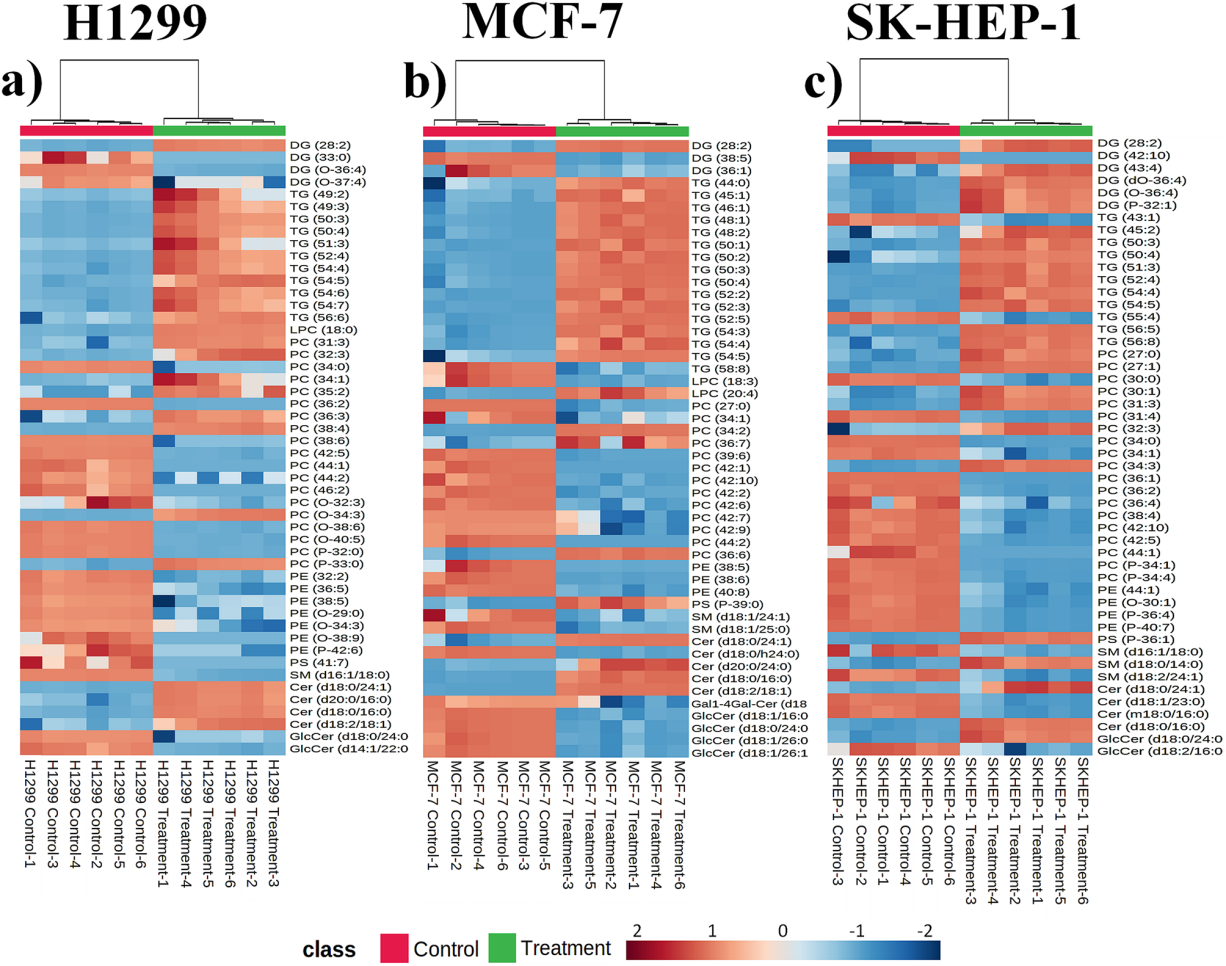
Heat map analysis of the extracted non-polar metabolomes of H1299 (**a**), MCF-7 (**b**), and SK-HEP-1 (**c**) cells analyzed in positive ionization mode. Groups are indicated at the top of each map by red (control, n = 6) and green (treatment [IC_50_ R-S5-C1-H1, 48 h], n = 6). The maps show 50 of the most significantly perturbed non-polar metabolites based on Euclidean distances and Ward clustering. The rows represent metabolites, while the columns represent the samples. The metabolite concentrations are represented on a log scale. The scale bar represents the normalized intensity of features. Decreased and increased metabolites are displayed in blue and red, respectively.

**Figure 7:**
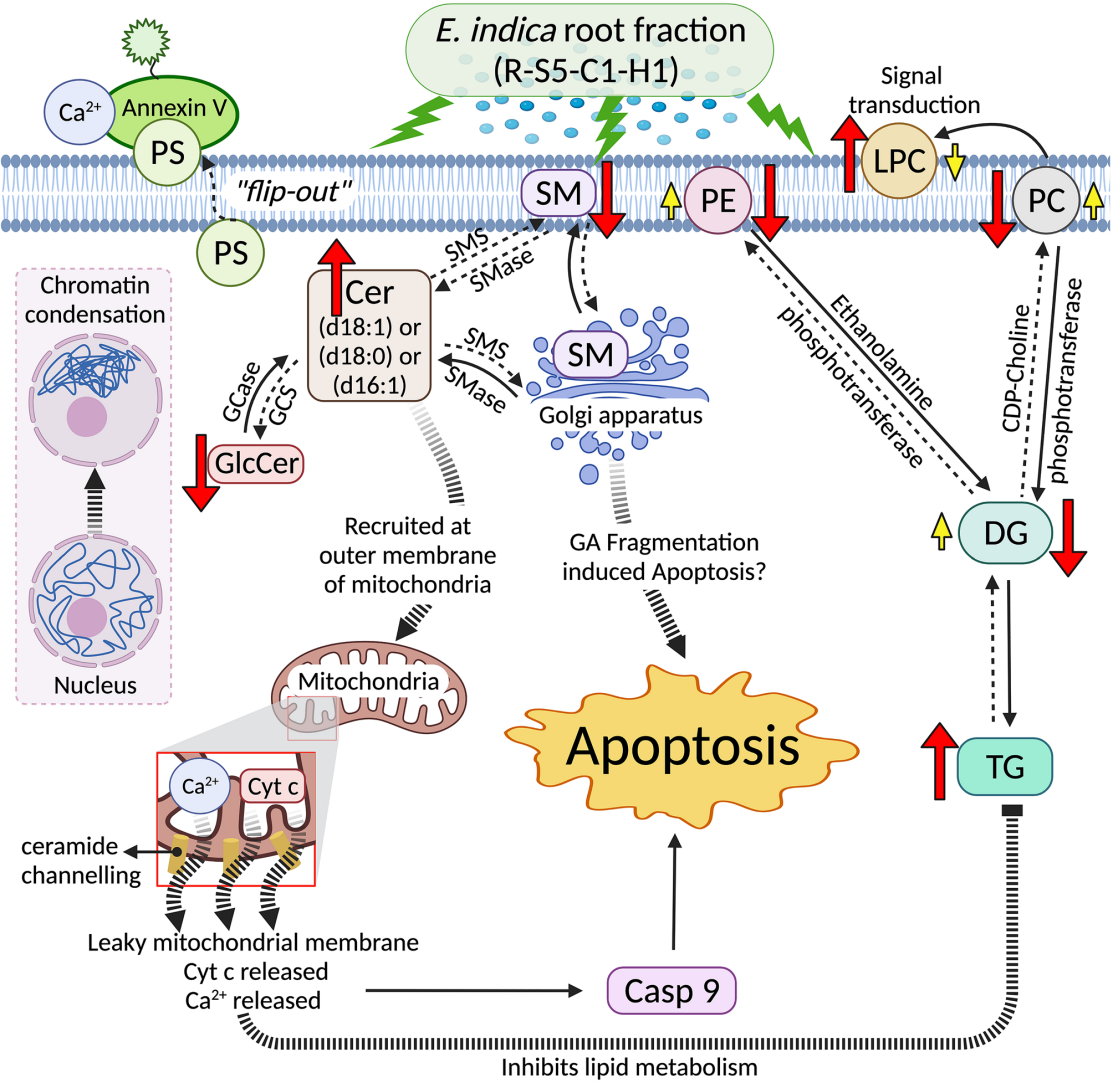
Overview of the metabolic pathways in cell lines 48 h after treatment with R-S5-C1-H1 *E.indica* root fraction. *SM* sphingomyelin, *PC* phosphatidylcholine, *LPC* lysophosphatidylcholine, *PE* phosphatidylethanolamine, *PS* phosphatidylserine, *TG* triacylglyceride, *DG* diacylglyceride, *Cer* ceramide, *GlcCer* glucosylceramide. The figure was created with BioRender.com.

Principal component analysis (PCA), an unsupervised pattern-recognition technique, was used to inspect data distribution and identify outliers throughout the analysis. For non-polar metabolomes profiled in positive ionization mode, PCA revealed distinct differences between treatment and control groups for H1299, MCF-7, and SK-HEP-1 cells after 48 h treatment with the IC_50_ of R-S5-C1-H1 (Fig. 5a–c i). However, in negative ionization mode, the metabolomes of the treatment and control groups of the three cell lines profiled were co-clustered, indicating that the profiled metabolomes showed no significant differences. Therefore, these data were excluded from the analysis (data not shown). Next, the cell metabolites that showed the greatest changes after R-S5-C1-H1 treatment were identified through the use of S-plots (Fig. 5a–c ii). The S-plots were constructed by comparing the treatment and control groups of each cell line via orthogonal projection to latent structure-discriminant analysis (OPLS-DA). The values of parameters R^2^Y and Q^2^ were used to describe the quality of the model, representing the model’s goodness-of-fit (R^2^Y) and predictability (Q^2^). The parameters of each model are listed in Supplementary Table S1. In each of the S-plots, the scatter plot p[1] represents the magnitude of each variable within a model, while p(corr)[1] represents the reliability of each variable (modeled correlation). The most significantly altered variables are plotted at either the lower left or upper right of the S-plot, and both are highly correlated with group separation. In this study, compounds at the lower left and upper right of the S-plots were selected as potentially relevant metabolites that were altered as a result of R-S5-C1-H1 treatment, based on a p(corr)[1] cutoff value of 0.8. These compounds are labeled as per Fig. 5a–c ii. The validity of the OPLS-DA model was demonstrated by a permutation test (Figure S2). S-plots (Fig. 5a–c ii) and heat map analysis (Fig. 6) revealed differing metabolomic profiles for control and treatment groups, suggesting that R-S5-C1-H1 altered the levels of non-polar (mainly lipid) metabolites in the cells. Even between the cancer cell lines, R-S5-C1-H1 affected the metabolites differently. In general, membrane lipids, such as phosphatidylcholines (PC), phosphorylethanolamines (PE), and sphingomyelins (SM) were dysregulated in cells treated with the IC_50_ of R-S5-C1-H1 for 48 h. In contrast, triacylglycerols (TGs), the main lipids used for energy storage in cells, and ceramide (Cer), an apoptosis inducer, were found to be upregulated in the treated H1299, MCF-7, and SK-HEP-1 cells. Details of the identified hydrophobic metabolites affected by treatment with R-S5-C1-H1 are summarized in Tables S4–S6. An overview of metabolic pathways in the R-S5-C1-H1-treated cell lines is shown in Fig. 7.

## Discussion

Secondary plant metabolites consist of numerous chemical compounds, including phenolics, alkaloids, saponins, terpenes, lipids, and complex carbohydrates, and are produced by plant cells via various metabolic pathways. These pathways are derived from primary metabolic pathways and involve secondary modifications such as deamination. Secondary metabolites have been reported to exert antibiotic, antifungal, and antiviral activities in order to protect plants from a wide range of pathogenic invasions. These compounds also possess other important biological properties, including anti-inflammatory, antibacterial, antioxidant, and selective anticancer^30,36^activities, which provide a scientific basis for the use of medicinal plants in traditional medicine.

In this study, the effect of the most active fraction of *E. indica* root extract, R-S5-C1-H1, on the viability of H1299, MCF-7, and SK-HEP-1 cells was evaluated using MTT assays. R-S5-C1-H1 was found to affect the viability of all the studied cancer cell lines in a dose- and time-dependent manner, in agreement with other studies investigating the anticancer activity of *E. indica*^33,34^ in different cell lines. At 72 h post-treatment, R-S5-C1-H1 IC_50_ values were below 20 µg/mL for all three cancer cell lines. This suggests that R-S5-C1-H1 was highly active against the tested cell lines according to the criteria established by the US National Cancer Institute and Geran protocol (where IC_50_ ≤ 20 µg/mL = highly active, IC_50_ 21–200 µg/mL = moderately active, IC_50_ 201–500 µg/mL = weakly active and IC_50_ ≥ 501 µg/mL = not active^37^). Cell viability was significantly reduced by higher concentrations of R-S5-C1-H1 or by longer treatment times. Prolonging treatment time while reducing drug dose can be a useful way of potentially minimizing undesirable side effects. A similar approach was applied in a clinical study conducted by Bishnoi et al. (2017) on recurrent, advanced, and metastatic cancer; lowering the chemotherapeutic dosage but prolonging therapy to achieve the desired outcomes extended patient survival while reducing undesirable side effects including nausea, neuropathy, fatigue, sepsis, and thrombocytopenia^38^. Similar results have been reported for advanced cancers, with improved quality of life achieved by application of this therapeutic approach^39–41^.

Cancer is defined as dysregulated cell proliferation combined with suppressed programmed cell death^42^. Anticancer agents that can induce programmed cell death in cancer cells, particularly apoptosis, while minimizing unwanted effects on the surrounding normal cells have received considerable attention because they offer a promising strategy for cancer prevention and treatment^43^. The majority of chemotherapy drugs currently in clinical use inhibit cell growth and induce apoptosis^44^. Cellular apoptosis is characterized by a series of typical morphological events, including membrane blebbing, cell shrinkage, chromatin condensation, nuclear fragmentation, fragmentation into membrane-bound apoptotic bodies, and translocation of membrane PS^45,46^. Microscopy analysis in the present study showed chromatin condensation, nuclear fragmentation, and binding of Annexin V dye to PS on the outer leaflet of the plasma membrane in the treated cells, suggesting that R-S5-C1-H1 was able to induce apoptotic cell death. However, further studies are needed to elucidate the underlying mechanism of apoptosis induced by *E. indica*.

According to the metabolome analysis in this study, several important non-polar lipid metabolites in the cancer cells were affected by treatment with R-S5-C1-H1. However, during polar metabolome extraction, residues from the culture medium (consisting of polar compounds, nutrients, and amino acids) become included during the process of snap freezing and extraction. Although concentrations of these residues may be low, they can introduce significant artifacts into polar metabolome profiling at the cellular level. Therefore, for polar metabolites, differences between control and *E. indica*-treated groups may be highly contributed by the culture medium instead of the responding metabolome. In negative ionization mode, co-clustering of the non-polar metabolomes of the treatment and control groups profiled was observed, indicating that the profiled metabolomes were not significantly different. Thus, in the current study, we focused on the non-polar metabolome in positive ionization mode. Lipids are small molecules with inherent hydrophobic properties. These molecules form key components of membranes, act as energy supplements, and play roles in inter- and intracellular signaling as well as in metabolic regulation. A mixture of complex lipids such as TGs, diacylglycerols (DGs), and monoacylglycerols (MGs), which consist of fatty acyl groups (varying in length and degree of unsaturation) esterified to glycerol backbones, is present in eukaryotic cells. During untargeted non-polar extract profiling using high-resolution mass spectrometry, TGs, DGs, and MGs can be distinguished from other lipid classes using positive electrospray ionization by the formation of ammoniated adduct ions, i.e., [M^+^ NH4]^+^ ions^23,47^. Collision-induced dissociation (CID) of the ammoniated ions yields an abundance of product ions corresponding to the loss of ammonia plus each of the fatty acyl groups as a free carboxylic acid^47,48^.

From our analysis, cellular energy homeostasis was suspected to be disrupted, as TGs were increased significantly. In vivo, it is possible to determine dysregulation of energy metabolism by analysis of glucose, pyruvate, and lactate concentrations in tissue. However, in vitro, monitoring of cultured cells based on these polar metabolites tends to be challenging as minute metabolite changes can be obscured by nutrients or supplements in the culture media, leading to false-positive results. Accumulation of TGs has been associated with cellular stress, including apoptosis^49^. Li et al. (2018) suggested that cells undergoing chemically-induced oxidative stress or apoptosis utilize TGs (with polyunsaturated fatty acyl chains) to protect against toxicity and limit cell death^49^. This phenomenon could explain the upregulation of TGs in all three cancer cell lines following treatment with R-S5-C1-H1 in the present study. Mitochondrial dysfunction and inhibition of lipid biosynthesis eventually lead cells to undergo apoptosis^50^.

Diacylglycerols play important roles as secondary messengers, activating proteins involved in various signaling cascades; thus, accumulation in biological systems is strictly regulated^51^. Phosphorylation of DGs to form phospholipids via diacylglycerol kinase leads to the attenuation of DG levels in the cell membrane, altering the availability of intracellular DG signaling protein and regulating cell growth, trafficking, differentiation, and migration^51^. During phospholipid synthesis, phosphocholine/phosphoethanolamine is transferred from cytidine diphosphate (CDP)-choline/ethanolamine to DGs by phosphotransferase, producing phosphatidylcholine (PC)/phosphatidylethanolamine (PE)^52^. Phosphatidylcholines account for nearly 50% of glycerophospholipid species in eukaryotic cells. The abundance of these phospholipids highlights their importance in biological and cellular systems as either membrane bilayer constituents or secondary messengers. Phosphorylcholine-containing lipid species are distinguished from other phospholipid classes using electrospray positive ionization, whereby CID of the protonated ions yields an abundant product ion, *m/z* 184, corresponding to a phosphorylcholine head group bonded at the sn-3 position of the glycerol backbone and fatty acyl substituents at sn-1 and/or sn-2^23,53^. Meanwhile, PE, an aminophospholipid, is the second most abundant phospholipid in mammalian membranes after PC^54^. It is concentrated on the cytosol-facing leaflet of the cell membrane, whereas phosphorylcholine-containing lipids (including PC and SM) are localized in the outer plasma membrane^55^. Phosphatidylethanolamine plays an important role in post-translational modifications, influencing membrane topology and promoting cell and organelle membrane fusion, oxidative phosphorylation, mitochondrial biogenesis, and autophagy^56^. Phosphorylethanolamine species are distinguished from other phospholipid classes by CID of the protonated ions, yielding an abundant daughter ion, [M^+^ H-141]^+^, with neutral loss of *m/z* 141, corresponding to the phosphorylethanolamine head group bonded at the sn-3 position^57^. Inhibition of the CDP-choline pathway is a common feature of apoptosis^58^, while perturbation of PC levels leads to the induction of apoptotic cell death irrespective of the PC synthesis steps that are impaired^59^. From our analysis, levels of DG, PC, and PE were significantly perturbed in the treated cells, suggesting that R-S5-C1-H1 might induce apoptosis by modifying phospholipid synthesis.

Sphingolipids, amphipathic lipids with diverse molecular structures and functions, act as key regulators in cancer cell survival and death^60^. These molecules all contain a long-chain (sphingoid) base (aliphatic amino alcohol) consisting of a long hydrocarbon tail with hydroxyl groups at sn-1 and sn-3 positions and an amine group at sn-2. The functions attributed to bioactive sphingolipids include cell growth, cell death, senescence, inflammation, immune responses, nutrient uptake, metabolism, responses to stress stimuli, and autophagy^61,62^. Collision-induced dissociation of protonated Cer species produces a unique fragment, *m/z* 264, corresponding to the sphingosine backbone (d18:1), whereas dihydroceramide (d18:0) and sphingadienine (d18:2) contribute fragments of *m/z* 266 and 262, respectively.

Ceramides are central molecules in sphingolipid metabolism and are composed of a sphingosine backbone and a long fatty acid chain (varying in length from C14 to C26) amide-bonded to the second carbon of the backbone. They serve as metabolic and structural precursors for complex sphingolipids (which contain hydrophilic head groups) such as SM, ceramide-1-phosphate, and carbohydrate-conjugated Cers^63^. Regulation of Cer is maintained by complex and integrated metabolic pathways involving specialized enzymes^63^. Sphingomyelinase (SMase) is an enzyme that hydrolyzes SM to yield Cer in the cytoplasm and Golgi apparatus respectively^64^, while the choline head group becomes bonded to DG to form PC. Cer has been reported to be synthesized de novo from the common sphinganine (d18:0) backbone^65^. The unsaturated sphingosine backbone (d18:1 or d18:2) enhances intermolecular hydrogen bonding in the polar region, tightening Cer molecule packing in the cellular membrane^62^. Importantly, Cer has the capability to assemble channels in the outer mitochondrial membranes to promote the release of cytochrome C for caspase-9 activation, leading to apoptosis^66,67^. It has been established that Cer is proapoptotic, with its generation preceding the onset of apoptotic signaling^68^. Many apoptotic stimuli, including cellular stress and cytotoxic drugs, have been shown to increase the levels of endogenous Cer through multiple mechanisms^68^, including inhibition of Cer metabolism enzymes^69^. In contrast, SM is mainly found in the Golgi apparatus^70^ due to the transport of Cer from the endoplasmic reticulum^71^. Upon arrival in the Golgi apparatus, Cer is converted into SM via the enzyme sphingomyelin synthase (SMS), which can induce cell proliferation, migration, and survival^72^. Metabolism of SM plays a role in cancer progression, whereby the breakdown of SM directly results in Cer production^73^. In addition, glucosylceramide (GlcCer), a pro-survival and antiapoptotic molecule generated from the transfer of glucose to Cer by glucosylceramide synthase (GCS), has been shown to be associated with multi-drug resistance in cancer cells^69^. Cers can be also produced from the degradation of GlcCers by the enzyme glucocerebrosidase (GCase)^74^. It has been suggested that the intracellular pool of Cer affects the pro-proliferative and antiapoptotic effects of GlcCer, whereby an increase in the synthesis of GlcCer decreases the level of Cer in cells^75^. In this study, the level of Cer was generally increased in the R-S5-C1-H1-treated cancer cells compared to controls, whereas levels of SM and GlcCer were decreased following treatment. These results suggest that R-S5-C1-H1 may induce apoptosis in the studied cancer cells by perturbing the balance of key sphingolipids through effects on sphingolipid metabolism.

In conclusion, R-S5-C1-H1 affected the viability of H1299, MCF-7, and SK-HEP-1 cells in a dose- and time-dependent manner. It also induced apoptotic cell death in the treated cells. Metabolomics based on LC–MS demonstrated that R-S5-C1-H1 caused different metabolic responses related to damage and rejuvenation in the cancer cell lines tested. Upregulation of TGs suggests protection of cells against R-S5-C1-H1 exposure, while perturbation of PC and PE levels may indicate induction of apoptosis by R-S5-C1-H1 through modulation of phospholipid synthesis. Dysregulated SM and increased levels of Cer species also suggest initiation of apoptosis, as these amphipathic lipids are responsible for the release of proapoptotic proteins from the mitochondria. Therefore, through high-throughput, unbiased profiling, we were able to screen for differences in multiple metabolites simultaneously. This method has advantages over conventional methods of analyzing the effects of therapeutic interventions, as it can facilitate the overall understanding of molecular events.

## Methods

### Plant collection

Wild *E. indica* was collected from the lowlands of Kota Belud (latitude 6.290833°N and longitude 116.424722°E), Sabah, Malaysia, in accordance with the Sabah Wildlife Conservation Enactment 1997. The plant was identified by Mr. Julius Kulip from the Institute for Tropical Biology and Conservation, Universiti Malaysia Sabah, and a voucher specimen (BORH 2263) was deposited at the Borneensis Herbarium of the Institute for Tropical Biology and Conservation, Universiti Malaysia Sabah, Kota Kinabalu, Malaysia.

### Crude sample preparation

The collected plant samples were thoroughly cleaned and stored at -20 °C prior to being freeze-dried using a Labconco freeze-dryer (Labconco, Kansas City, MO, USA). Freeze-dried *E. Indica* was ground using a heavy-duty blender. The homogenized sample was then exhaustively extracted using a modified extraction method^23^, whereby an optimized ratio of 1:1:1 (v/v/v) of double-distilled water (Milli-Q system (Merck, Darmstadt, Germany) at a resistivity of > 18.2 MΩ-cm/methanol (MeOH)/chloroform was employed. Following this, the lower layer of the mixture was removed and vacuum-concentrated to obtain a semisolid crude extract.

### Bioassay-guided fractionation

Crude extracts of the different plant parts (seeds, stem, leaf, and roots) were screened by being used to treat H1299, MCF-7, and SK-HEP-1 cell lines. Based on the crude extract screening (Fig. S1a), the root extract, which exhibited the highest activity against all cell lines, was selected for solid-phase extraction (SPE). An OASIS HLB Cartridge SPE column was used to purify the extract according to the solvent mixture stated in Table S2. From the SPE screening, R-S5 was the most active fraction (Fig. S1b); this was then subjected to normal-phase open-column chromatography in columns packed with silica gel with mesh size ranging between 0.063 and 0.200 mm (Merck, Darmstadt, Germany). Elution was initiated with hexane:ethyl acetate (9:1, v/v) and completed with MeOH (Table S3). The most active fraction, R-S5-C1 (Fig. S1c), was further separated using the Agilent 1200 series HPLC system (Agilent Technologies, Waldbronn, Germany) coupled with a Thermo Scientific Accucore C18 column (2.1 mm × 150 mm  ×  2.6 µm; Sunnyvale, CA, USA). Mobile phase A consisted of a mixture of deionized water with 0.1% formic acid and 1% ammonium acetate (NH_4_CH_3_CO_2_), while mobile phase B consisted of a mixture of acetonitrile and MeOH (6:4 v/v) with 0.1% formic acid and 1% NH_4_CH_3_CO_2_. The elution gradient was programmed to increase linearly from 1 to 70% of solvent B in 7 min, followed by 100% of solvent B from 7.1 to 10 min, maintained for 3 min. Later, the column was conditioned with the initial gradient for 1 min before the next sample injection. According to the results of the final screening, R-S5-C1-H1 was the most active fraction against all cell lines (Fig. S1d) and was therefore selected for further analyses. R-S5-C1-H1 was fully suspended in dimethylsulfoxide (DMSO), with a final concentration of DMSO ≤ 0.1% (v/v) prior to all cell experiments.

### Cell culture

Non-small cell lung carcinoma H1299 (ATCC CRL-5803™), hepatocellular carcinoma SK-HEP-1 (ATCC HTB-52™), and breast adenocarcinoma MCF-7 (ATCC HTB-22™) cell lines were purchased from the American Type Culture Collection (Manassas, VA, USA). SK-HEP-1 cells were maintained in Dulbecco’s Modified Eagle’s Medium, while the other cell lines were cultured in Roswell Park Memorial Institute 1640 medium. All media were supplemented with 10% fetal bovine serum (Tico Europe, Amstelveen, Netherlands) and 1% penicillin–streptomycin (Nacalai Tesque, Kyoto, Japan). The cells were grown in a humidified environment with 5% CO_2_ at 37 °C.

### Cell viability and half-maximal inhibitory concentration (IC_50_) determination

The viability of H1299, MCF-7, and SK-HEP-1 cells was assessed using the colorimetric [3-(4,5-Dimethylthiazol-2-yl)-2,5-diphenyltetrazolium bromide] (MTT) assay (Nacalai Tesque, Kyoto, Japan). Briefly, cells were seeded at 5  ×  10^3^ cells/well (100 μL) on a 96-well plate (Nunc, Thermo Fisher Scientific, Denmark) and incubated for 24 h to allow cell attachment. After 24 h, the cells were treated with varying concentrations of R-S5-C1-H1, ranging from 0.05 to 100 μg/mL, for 24 h, 48 h, and 72 h. Doxorubicin (0.01 to 100 μg/mL) served as a positive control. The formazan precipitate in each well was suspended in DMSO, and absorbance was measured at 570 nm using the Tecan Infinite 200 Pro microplate reader (Männedorf, Switzerland). Percentage cell viability (%) was calculated as $\frac{Absorbance\, of\, sample}{Absorbance\, of\, control }\times 100\%$. All experiments were performed in triplicate, and data were presented as mean ± standard error of the mean (SEM). Statistically significant differences at p ≤ 0.05 significance level were determined using the t-test (*p ≤ 0.05; **p ≤ 0.001).

### Microscopic observation of cell morphology

All cells were seeded at 2.5  ×  10^5^ cells/well (500 µL) in a 4-well µ-slide (ibidi, Martinsried, Germany) and incubated overnight before being treated with the IC_50_ of R-S5-C1-H1 for 48 h. Treated cells were fixed and stained concurrently with Hoechst 33342 at 1 μg/mL (Sigma, USA) and Annexin V at 10 μg/mL (Nacalai Tesque, Kyoto, Japan) at 37 °C for 30 min in the dark. Morphological observations were performed using a fluorescence microscope, BX 53 (Olympus, Tokyo, Japan), to visualize nuclear condensation and PS externalization with ultraviolet and green fluorescence filters, respectively (Olympus, Tokyo, Japan).

### Cell metabolome extraction for LC–MS/MS analysis

Each cell line was seeded at 1  ×  10^6^ cells/dish in 12 culture dishes. Six dishes of each cell line were treated with the IC_50_ of R-S5-C1-H1 for 48 h, while the remaining six dishes served as untreated controls. The culture medium from each culture dish was collected and centrifuged at 1500 g to acquire a cell pellet. Adhered cells were snap-frozen with pre-cooled HPLC grade MeOH, collected by scraping, and then transferred into clean disposable borosilicate tubes together with the cell pellets from the previous step. The combined cells were extracted using a modified Bligh and Dyer extraction protocol^65^. Briefly, cells were mixed thoroughly with 4 mL MeOH/chloroform (1:1 v/v), followed by 2 mL of 0.05 M sodium chloride, and centrifuged at 1500 g at 4 °C for 30 min. Both polar and non-polar of cell extracts were transferred separately and vaporized using a speed vacuum concentrator system (Eppendorf, Hamburg, Germany) before being stored at -80 °C. Prior to LC–MS/MS analysis, the cell extracts were redissolved in 1.2 mL MeOH. Each cell extract was profiled in triplicate to ensure the consistency of the instrument.

### LC–MS/MS

Cell extract profiling was carried out according to a previously developed method^76,77^. Briefly, 10 μL or 30 μL of extract were applied to a Vanquish UHPLC system (Thermo Scientific, Waltham, MA, USA) coupled to the ultra-high resolution Qq-Time-of-flight Impact II mass spectrometer (Bruker, Billerica, MA, USA) in positive and negative electrospray ionization modes, respectively. A pentafluorophenyl column, Kinetex F5 (2.1 mm × 100 mm × 2.6 µm; Phenomenex, Torrance, California, USA), was used for chromatographic separation at 35 °C and the mobile phase flow rate was maintained at 0.6 mL/min. Mobile phase A consisted of a mixture of deionized water with 0.1% formic acid and 1% NH_4_CH_3_CO_2_, and mobile phase B consisted of a mixture of acetonitrile and MeOH (6:4 v/v) with 0.1% formic acid and 1% NH_4_CH_3_CO_2_. The gradient elution was programmed to increase linearly from 1 to 70% of solvent B in 7 min, followed by 100% solvent B from 7.1 to 10 min, maintained for 3 min. Following this, the column was conditioned with the initial gradient for 1 min before the next sample was introduced. Data acquisition was set between *m/z* 50 and 1500, while positive and negative electrospray ionization voltages were set at 3.5 kV and − 3.5 kV, respectively. Ion source gas temperature was set at 325 °C, drying gas flow at 10 L/min, and nebulizer flow at 3 Bar. The mass spectrometer was calibrated with Tune Mix (Sigma-Aldrich, St Louis, MO, USA) before analysis of each batch. A mass calibrant, sodium formate, was introduced between 0.1 and 0.3 min during each acquisition. Post-acquisition analyte *m/z* values were calibrated against the introduced sodium formate.

### Chemometric analysis

Acquired raw data were pre-processed using MZmine 2^35^ to compensate for the variation in retention times and *m/z* values in each analysis. These pre-process data were exported as a peak list in comma-separated values (CSV) format, with rows representing the integrated and normalized peak areas and columns representing the samples. Exported .csv files were used for multivariate analyses with Metaboanalyst 5.0 (version 5.0; http://www.metaboanalyst.ca)^78^. Metabolite features with missing values > 50% were removed, and missing values were replaced by 1/5 of minimum positive values of the corresponding variables by default. The acquired data sets were log-transformed and Pareto-scaled prior to multivariable analyses to examine data distribution and identify outliers. Multivariate analyses of data including PCA, OPLS-DA, and hierarchical cluster analysis (heat map) were obtained. Subsequently, for OPLS-DA, the model examined internal relationships in matrix X-variables and response matrix Y. Model quality was estimated by R^2^X or R^2^Y values and Q^2^ values. To avoid OPLS-DA model over-fitting, an additional cross-validation tool (permutation test with Y variables randomized 100 times) was performed for each model. Goodness-of-fit parameters for the OPLS-DA model, R^2^X, R^2^Y, and Q^2^ were calculated (varying from 0 to 1). The R^2^Y and Q^2^ in the permutated test described the fitness of the data and the predictability of the derived model, respectively. In addition, the metabolite features (non-parametric t-test, *p* < 0.05) and corresponding fold changes (fold change threshold ≥ 2) showed how the selected differential metabolites varied between the treatment and control groups; these metabolites were selected and underwent further compound matching and analysis. In the current study, all metabolomics data analyses focused on the non-polar layer only and were performed on the positive ionization mode data sets, as the negative ionization mode revealed no significant differences (data excluded).

### Metabolite identification and statistical analysis

Based on the normalized acquired data, statistical significance was determined by t-tests (non-parametric analysis). Identification of metabolites was based on ion fragmentation pattern recognition^23,79^ and fragmentation spectra (MS/MS) matching using Metfrag^80^, Human Metabolome Database (HMDB)^81^ and Lipid Metabolites and Pathway Strategy (LIPIDMAPS)^82^. Mass-to-charge ratios complemented with fragmentation spectra and acceptable mass tolerance (at 5 ppm) revealed the metabolome identity. Furthermore, determination of lipid molecular formulae was achieved via the description of fatty acid substituents through mathematical calculation of mass balance for molecular weight based on high-resolution mass accuracy.

## Supplementary Information

Supplementary Information.

## Data Availability

All data generated or analysed during this study are included in this published article (and its Supplementary Information file). Additional raw data files can be available from the corresponding authors upon reasonable request.
