# Supplementary material for: High-throughput metabolomics reveals dysregulation of hydrophobic metabolomes in cancer cell lines by Eleusine indica
Source: Sci Rep. 2022 Jun 6;12:9347. doi: 10.1038/s41598-022-13575-6 (PMC9168358; doi:10.1038/s41598-022-13575-6)
Supplement: Supplementary file 1 — Supplementary Information. [file 41598_2022_13575_MOESM1_ESM.docx]

**Supplementary information**

**
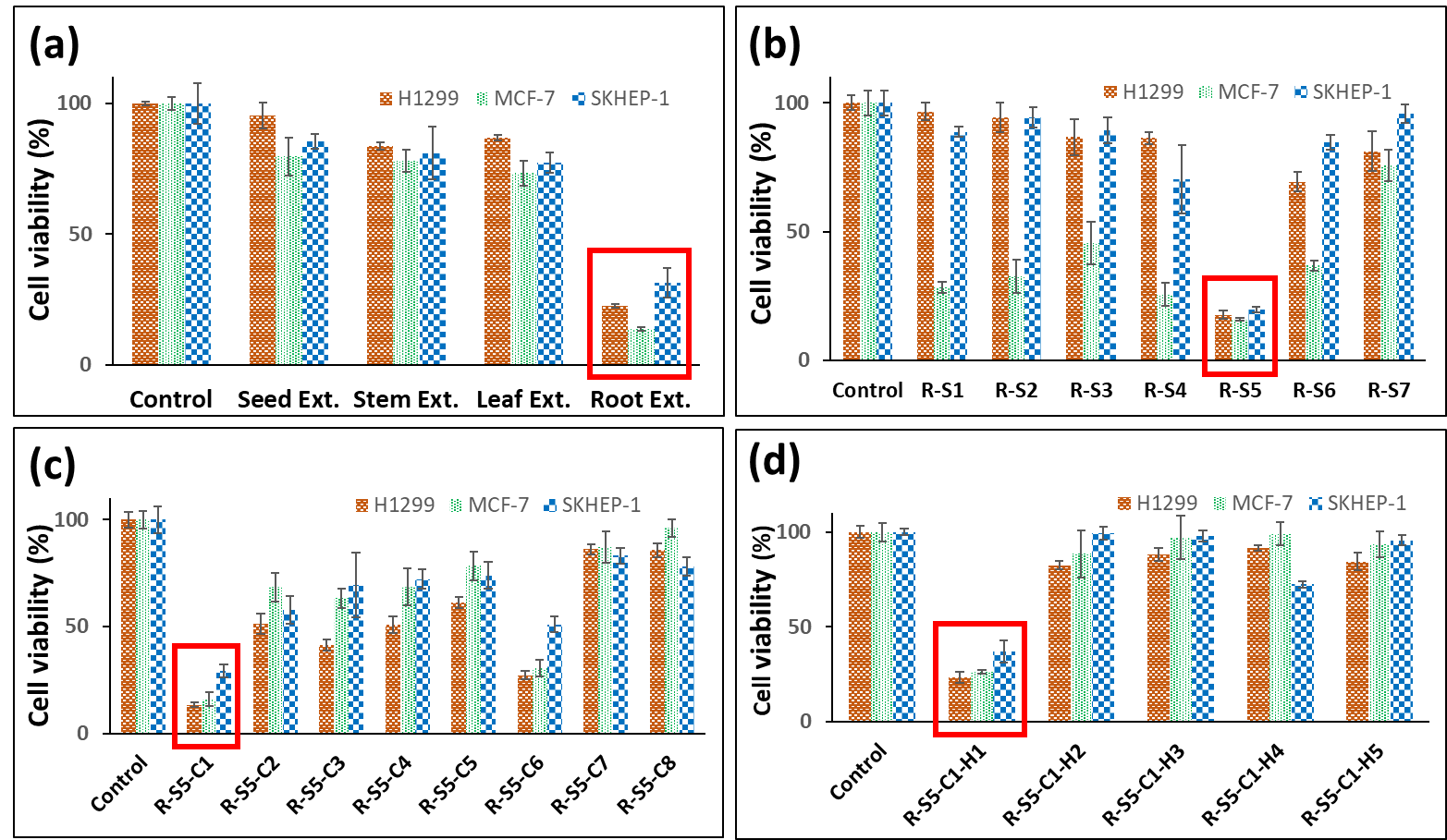
**

**Supplementary Figure S1.** Preliminary screening of the most effective anti-cancer isolates from *E. indica.* against various cancer cells line assessed by MTT assay. (a) Crude extract from different parts of *E. indica.* screen at 200 µg/mL for 72 h, (b) Solid-phase extraction (SPE) fractions screen at 100 µg/mL for 72 h, (c) Open column chromatography (CC) fractions screen at 100 µg/mL for 24 h, (d) HPLC isolates screen at 100 µg/mL for 12 h. *Most effective anti-cancer fraction/isolates (red box) was selected for next study. Values represent mean ± SD of three independent experiments.

**Supplementary Figure S2.** The response OPLS-DA permutation test result of non-polar metabolomes of (a) H1299, (b) MCF-7, and (c) SK-HEP-1 cells obtained from 100 tests.

**Supplementary Table S1. Parameters of OPLS-DA models derived from metabolites data sets of hydrophobic metabolites data sets of three different cancer cells line.**

|  | Treatment vs Control | | |
| --- | --- | --- | --- |
|  | H1299 | MCF-7 | SK-HEP-1 |
| R^2^Y | 0.994 | 0.998 | 0.999 |
| Q^2^ | 0.979 | 0.988 | 0.984 |

**Supplementary Table S2. Solvent ratio applied on solid-phase extraction chromatography at different isolate fractions.**

| **Fraction** | **Code** | **Solvent mixture** | **Ratio (v/v)** | **Total Volume (mL)** |
| --- | --- | --- | --- | --- |
| 1 | R-S1 | Water : methanol | 75:25 | 5 |
| 2 | R-S2 | Water : methanol | 50:50 | 5 |
| 3 | R-S3 | Water : methanol | 25:75 | 5 |
| 4 | R-S4 | Methanol | - | 5 |
| 5 | R-S5 | Methanol : ethyl acetate | 75:25 | 5 |
| 6 | R-S6 | Methanol : ethyl acetate | 50:50 | 5 |
| 7 | R-S7 | Methanol : ethyl acetate | 25:75 | 10 |

**Supplementary Table S3. Solvent ratio applied on open-column chromatography (column 25 × 250 mm) at different isolate fractions.**

| **Fraction** | **Code** | **Solvent mixture** | **Ratio (v/v)** | **Total Volume (mL)** |
| --- | --- | --- | --- | --- |
| 1 | R-S5-C1 | Hexane : ethyl acetate | 75:25 | 50 |
| 2 | R-S5-C2 | Hexane : ethyl acetate | 50:50 | 50 |
| 3 | R-S5-C3 | Hexane : ethyl acetate | 25:75 | 50 |
| 4 | R-S5-C4 | Ethyl acetate | - | 50 |
| 5 | R-S5-C5 | Ethyl acetate: methanol | 75:25 | 50 |
| 6 | R-S5-C6 | Ethyl acetate : methanol | 50:50 | 50 |
| 7 | R-S5-C7 | Ethyl acetate: methanol | 25:75 | 50 |
| 8 | R-S5-C8 | Methanol | - | 100 |

**Supplementary Table S4. Significant changes of hydrophobic metabolites identified from H1299 cells induced by 48 h treatment with *E. indica* root fraction (R-S5-C1-H1) using LC-MS/MS.**

| **Lipid Class** | **Detected m/z** | **Ion Adduct** | **Lipids Identification^a^** | ***p*-value^b^** | **Fold-changes^c^** |
| --- | --- | --- | --- | --- | --- |
| **a. Glycerolipids** |  |  |  |  |  |
| Diacylglycerol (DG) | 509.4184 | [M+H]^+^ | DG (28:2) | ●●● | ↑↑↑↑↑ |
|  | 551.5044 | [M+H]^+^ | DG (P-32:1) | ●●● | ↑↑ |
|  | 558.5103 | [M+NH_4_]^+^ | DG (30:0) | ● | ↑↑ |
|  | 583.5308 | [M+H]^+^ | DG (33:0) | ●●● | **↓↓↓↓↓** |
|  | 589.5566 | [M+H]^+^ | DG (dO-36:4) | ●●● | ↑↑↑ |
|  | 603.5358 | [M+H]^+^ | DG (O-36:4) | ●●● | **↓↓↓↓↓** |
|  | 617.5509 | [M+H]^+^ | DG (O-37:4) | ●● | **↓↓↓↓** |
|  | 624.5576 | [M+NH_4_]^+^ | DG (35:2) | ● | ↑ |
|  | 662.5735 | [M+NH_4_]^+^ | DG (38:4) | ●●● | ↑ |
| Triacylglycerols (TG) | 768.7089 | [M+NH_4_]^+^ | TG (44:0) | ●●● | ↑ |
|  | 818.7249 | [M+NH_4_]^+^ | TG (48:3) | ●●● | ↑↑ |
|  | 820.7404 | [M+NH_4_]^+^ | TG (48:2) | ●● | ↑↑↑↑ |
|  | 832.7403 | [M+NH_4_]^+^ | TG (49:3) | ●●● | ↑↑↑↑ |
|  | 834.7561 | [M+NH_4_]^+^ | TG (49:2) | ●● | ↑↑↑↑↑ |
|  | 836.7724 | [M+NH_4_]^+^ | TG (49:1) | ●●● | ↑↑↑ |
|  | 844.7404 | [M+NH_4_]^+^ | TG (50:4) | ●●● | ↑↑↑↑↑ |
|  | 846.7561 | [M+NH_4_]^+^ | TG (50:3) | ●●● | ↑↑↑↑↑ |
|  | 848.7717 | [M+NH_4_]^+^ | TG (50:2) | ●●● | ↑ |
|  | 850.7878 | [M+NH_4_]^+^ | TG (50:1) | ●●● | ↑ |
|  | 860.7718 | [M+NH_4_]^+^ | TG (51:3) | ●●● | ↑↑↑↑↑ |
|  | 862.7877 | [M+NH_4_]^+^ | TG (51:2) | ●● | ↑↑↑↑↑ |
|  | 870.7554 | [M+NH_4_]^+^ | TG (52:5) | ●● | ↑↑↑ |
|  | 872.7715 | [M+NH_4_]^+^ | TG (52:4) | ●●● | ↑↑↑↑ |
|  | 874.7872 | [M+NH_4_]^+^ | TG (52:3) | ●●● | ↑↑ |
|  | 876.8035 | [M+NH_4_]^+^ | TG (52:2) | ●● | ↑↑ |
|  | 884.7737 | [M+NH_4_]^+^ | TG (53:5) | ● | ↑ |
|  | 894.7563 | [M+NH_4_]^+^ | TG (54:7) | ●●● | ↑↑↑↑ |
|  | 896.7716 | [M+NH_4_]^+^ | TG (54:6) | ●●● | ↑↑↑↑↑ |
|  | 898.7868 | [M+NH_4_]^+^ | TG (54:5) | ●●● | ↑↑↑↑↑ |
|  | 900.8030 | [M+NH_4_]^+^ | TG (54:4) | ●●● | ↑↑↑↑ |
|  | 902.8192 | [M+NH_4_]^+^ | TG (54:3) | ●●● | ↑ |
|  | 920.7723 | [M+NH_4_]^+^ | TG (56:8) | ●●● | ↑↑↑ |
|  | 922.7875 | [M+NH_4_]^+^ | TG (56:7) | ●●● | ↑↑ |
|  | 924.8062 | [M+NH_4_]^+^ | TG (56:6) | ●●● | ↑↑↑ |
|  | 926.8183 | [M+NH_4_]^+^ | TG (56:5) | ●● | ↑↑↑ |
|  | 948.8033 | [M+NH_4_]^+^ | TG (58:8) | ●●● | ↑↑ |
|  | 950.8185 | [M+NH_4_]^+^ | TG (58:7) | ●● | ↑↑↑↑↑ |
| **b. Glycerophospholipids** |  |  |  |  |  |
| Lyso-Phosphatidylcholine (LPC) | 468.3094 | [M+H]^+^ | LPC (14:0) | ●●● | ↑ |
|  | 496.3409 | [M+H]^+^ | LPC (16:0) | ●●● | ↑↑ |
|  | 518.3226 | [M+H]^+^ | LPC (18:3) | ●●● | ↑↑↑ |
|  | 524.3720 | [M+H]^+^ | LPC (18:0) | ●●● | ↑↑↑ |
|  | 544.3409 | [M+H]^+^ | LPC (20:4) | ●●● | ↑↑↑ |
|  | 482.3626 | [M+H]^+^ | LPC (O-16:0) | ●●● | **↓↓** |
| Phosphatidylcholine (PC) | 662.4766 | [M+H]^+^ | PC (27:1) | ● | ↑↑↑ |
|  | 714.5085 | [M+H]^+^ | PC (31:3) | ●●● | ↑↑↑↑↑ |
|  | 720.5556 | [M+H]^+^ | PC (31:0) | ●●● | ↑↑↑ |
|  | 728.5236 | [M+H]^+^ | PC (32:3) | ●●● | ↑↑↑↑ |
|  | 734.5687 | [M+H]^+^ | PC (32:0) | ●●● | **↓↓** |
|  | 746.5709 | [M+H]^+^ | PC (33:1) | ● | **↓** |
|  | 748.5875 | [M+H]^+^ | PC (33:0) | ●●● | ↑↑ |
|  | 752.5231 | [M+H]^+^ | PC (34:5) | ●●● | ↑↑ |
|  | 756.5540 | [M+H]^+^ | PC (34:3) | ●● | ↑ |
|  | 758.5696 | [M+H]^+^ | PC (34:2) | ●● | ↑↑ |
|  | 760.5853 | [M+H]^+^ | PC (34:1) | ●●● | ↑↑↑↑↑ |
|  | 762.6011 | [M+H]^+^ | PC (34:0) | ●●● | **↓↓↓** |
|  | 772.5818 | [M+H]^+^ | PC (35:2) | ●●● | ↑↑↑↑↑ |
|  | 778.5398 | [M+H]^+^ | PC (36:6) | ●●● | ↑↑↑ |
|  | 782.5702 | [M+H]^+^ | PC (36:4) | ●●● | ↑↑↑ |
|  | 784.5837 | [M+H]^+^ | PC (36:3) | ●●● | ↑↑↑↑ |
|  | 786.6024 | [M+H]^+^ | PC (36:2) | ●●● | **↓↓↓↓↓** |
|  | 806.5675 | [M+H]^+^ | PC (38:6) | ●●● | **↓↓↓↓** |
|  | 810.6020 | [M+H]^+^ | PC (38:4) | ●●● | ↑↑↑↑ |
|  | 814.6337 | [M+H]^+^ | PC (38:2) | ●●● | **↓** |
|  | 820.5880 | [M+H]^+^ | PC (39:6) | ●●● | ↑↑ |
|  | 836.6159 | [M+H]^+^ | PC (40:5) | ●●● | **↓↓** |
|  | 842.6650 | [M+H]^+^ | PC (40:2) | ●●● | **↓↓** |
|  | 856.5845 | [M+H]^+^ | PC (42:9) | ● | **↓↓** |
|  | 860.6176 | [M+H]^+^ | PC (42:7) | ●●● | **↓** |
|  | 864.6491 | [M+H]^+^ | PC (42:5) | ●●● | **↓↓↓↓↓** |
|  | 870.6962 | [M+H]^+^ | PC (42:2) | ●●● | **↓↓** |
|  | 898.7273 | [M+H]^+^ | PC (44:2) | ●●● | **↓↓** |
|  | 900.7431 | [M+H]^+^ | PC (44:1) | ●●● | **↓↓** |
|  | 926.7588 | [M+H]^+^ | PC (46:2) | ●●● | **↓↓** |
|  | 714.5432 | [M+H]^+^ | PC (O-32:3) | ●● | **↓↓↓** |
|  | 720.5919 | [M+H]^+^ | PC (O-32:0) | ●● | **↓** |
|  | 742.5755 | [M+H]^+^ | PC (O-34:3) | ●●● | ↑↑↑↑ |
|  | 746.6051 | [M+H]^+^ | PC (O-34:1) | ● | **↓↓↓** |
|  | 792.5925 | [M+H]^+^ | PC (O-38:6) | ●●● | **↓↓↓** |
|  | 804.6855 | [M+H]^+^ | PC (O-38:0) | ● | ↑↑ |
|  | 822.6382 | [M+H]^+^ | PC (O-40:5) | ●●● | **↓↓↓** |
|  | 718.5762 | [M+H]^+^ | PC (P-32:0) | ●●● | **↓↓↓↓** |
|  | 732.5895 | [M+H]^+^ | PC (P-33:0) | ●●● | ↑↑↑ |
|  | 738.5453 | [M+H]^+^ | PC (P-34:4) | ●● | **↓↓** |
|  | 744.5918 | [M+H]^+^ | PC (P-34:1) | ●● | **↓↓↓↓** |
|  | 790.5753 | [M+H]^+^ | PC (P-38:6) | ●●● | ↑ |
| Phosphatidylethanolamine (PE) | 688.4941 | [M+H]^+^ | PE (32:2) | ●●● | **↓↓↓** |
|  | 692.5240 | [M+H]^+^ | PE (32:0) | ●●● | ↑ |
|  | 716.5239 | [M+H]^+^ | PE (34:2) | ●●● | **↓↓** |
|  | 738.5094 | [M+H]^+^ | PE (36:5) | ●●● | **↓↓↓↓** |
|  | 740.5237 | [M+H]^+^ | PE (36:4) | ●●● | **↓↓** |
|  | 764.5248 | [M+H]^+^ | PE (38:6) | ●●● | **↓** |
|  | 766.5387 | [M+H]^+^ | PE (38:5) | ●●● | **↓↓↓** |
|  | 790.5414 | [M+H]^+^ | PE (40:7) | ●●● | **↓** |
|  | 636.4965 | [M+H]^+^ | PE (O-29:0) | ●●● | **↓↓↓↓** |
|  | 700.5261 | [M+H]^+^ | PE (O-34:3) | ●●● | **↓↓↓** |
|  | 744.4961 | [M+H]^+^ | PE (O-38:9) | ●●● | **↓↓↓↓↓** |
|  | 748.5296 | [M+H]^+^ | PE (O-38:7) | ●●● | **↓↓** |
|  | 722.5113 | [M+H]^+^ | PE (P-34:2) | ●● | ↑ |
|  | 724.5287 | [M+H]^+^ | PE (P-36:4) | ●●● | **↓↓** |
|  | 774.5454 | [M+H]^+^ | PE (P-40:7) | ●●● | **↓↓** |
|  | 804.5882 | [M+H]^+^ | PE (P-42:6) | ●●● | **↓↓↓↓↓** |
| Phosphatidylserine (PS) | 788.5447 | [M+H]^+^ | PS (36:2) | ●●● | **↓↓** |
|  | 848.5472 | [M+H]^+^ | PS (41:7) | ●●● | **↓↓↓↓↓** |
| **c. Sphingolipids** |  |  |  |  |  |
| Sphingomyelin (SM) | 675.5450 | [M+H]^+^ | SM (d18:1/14:0) | ● | ↑↑↑↑↑ |
|  | 689.5605 | [M+H]^+^ | SM (d16:1/17:0) | ●●● | **↓** |
|  | 701.5606 | [M+H]^+^ | SM (d18:1/16:1) | ●●● | **↓** |
|  | 703.5754 | [M+H]^+^ | SM (d16:1/18:0) | ●●● | **↓↓↓↓** |
|  | 789.6810 | [M+H]^+^ | SM (d18:0/22:0) | ●●● | **↓** |
| Ceramide (Cer) | 540.5361 | [M+H]^+^ | Cer (d18:0/16:0) | ●●● | ↑↑↑↑↑ |
|  | 562.5195 | [M+H]^+^ | Cer (d18:2/18:1) | ●●● | ↑↑↑↑↑ |
|  | 568.5677 | [M+H]^+^ | Cer (d20:0/16:0) | ●●● | ↑↑↑↑ |
|  | 650.6439 | [M+H]^+^ | Cer (d18:0/24:1) | ●●● | ↑↑↑↑↑ |
|  | 668.6565 | [M+H]^+^ | Cer (d18:0/h24:0) | ●●● | ↑↑ |
|  | 700.5733 | [M+H]^+^ | GlcCer (d18:1/16:0) | ●●● | **↓↓** |
|  | 761.6267 | [M+NH_4_]^+^ | GlcCer (d14:1/22:0(2OH)) | ●●● | **↓↓↓** |
|  | 810.6829 | [M+H]^+^ | GlcCer (d18:1/24:1) | ●●● | **↓↓** |
|  | 812.6984 | [M+H]^+^ | GlcCer (d18:0/24:1) | ●●● | **↓↓** |
|  | 814.7134 | [M+H]^+^ | GlcCer (d18:0/24:0) | ●●● | **↓↓↓** |
|  | 862.6268 | [M+H]^+^ | Galα1-4Galβ-Cer (d18:1/16:0) | ●●● | ↑↑ |

^a^ Metabolites identification was matched with LIPIDMAPS based on its mass accuracy and fragmented spectral.

^b^ ●, *p*-value < 0.05; ●● *p*-value < 0.01; ●●● *p*-value < 0.001 determined by t-test (non-parametric analysis).

^c^ Fold changes of peak area relative to mean control. Log_2_ transformed. ↑, up-regulated ≥ 1 folds, ↑↑, up-regulated ≥ 2 folds; ↑↑↑, up-regulated ≥ 3 folds; ↑↑↑↑, up-regulated ≥ 4 folds; ↑↑↑↑↑, up-regulated ≥ 5 folds and *vice versa*.

**Supplementary Table S5. Significant changes of hydrophobic metabolites identified from MCF-7 cells induced by 48 h treatment with *E. indica* root fraction (R-S5-C1-H1) using LC-MS/MS.**

| **Lipid Class** | **Detected m/z** | **Ion Adduct** | **Lipids Identification^a^** | ***p*-value^b^** | **Fold-changes^c^** |
| --- | --- | --- | --- | --- | --- |
| **a. Glycerolipids** |  |  |  |  |  |
| Diacylglycerol (DG) | 509.4178 | [M+H]^+^ | DG (28:2) | ●●● | ↑↑↑↑↑ |
|  | 640.5884 | [M+NH_4_]^+^ | DG (36:1) | ●● | **↓** |
|  | 660.5569 | [M+NH_4_]^+^ | DG (38:5) | ●●● | **↓** |
| Triacylglycerols (TG) | 768.7087 | [M+NH_4_]^+^ | TG (44:0) | ●●● | ↑↑ |
|  | 780.7089 | [M+NH_4_]^+^ | TG (45:1) | ●●● | ↑↑↑↑↑ |
|  | 794.7244 | [M+NH_4_]^+^ | TG (46:1) | ●●● | ↑↑ |
|  | 818.7237 | [M+NH_4_]^+^ | TG (48:3) | ●●● | ↑ |
|  | 820.7400 | [M+NH_4_]^+^ | TG (48:2) | ●●● | ↑↑ |
|  | 822.7558 | [M+NH_4_]^+^ | TG (48:1) | ●●● | ↑↑ |
|  | 832.7398 | [M+NH_4_]^+^ | TG (49:3) | ●●● | ↑ |
|  | 834.7555 | [M+NH_4_]^+^ | TG (49:2) | ●●● | ↑ |
|  | 836.7717 | [M+NH_4_]^+^ | TG (49:1) | ●● | ↑ |
|  | 844.7400 | [M+NH_4_]^+^ | TG (50:4) | ●●● | ↑↑↑↑ |
|  | 846.7554 | [M+NH_4_]^+^ | TG (50:3) | ●●● | ↑↑↑ |
|  | 848.7712 | [M+NH_4_]^+^ | TG (50:2) | ●●● | ↑↑ |
|  | 850.7871 | [M+NH_4_]^+^ | TG (50:1) | ●●● | ↑↑ |
|  | 870.7555 | [M+NH_4_]^+^ | TG (52:5) | ●●● | ↑↑↑↑↑ |
|  | 872.7706 | [M+NH_4_]^+^ | TG (52:4) | ●●● | ↑ |
|  | 874.7868 | [M+NH_4_]^+^ | TG (52:3) | ●●● | ↑↑↑ |
|  | 876.8028 | [M+NH_4_]^+^ | TG (52:2) | ●●● | ↑↑ |
|  | 884.7734 | [M+NH_4_]^+^ | TG (53:5) | ●● | ↑↑ |
|  | 886.7862 | [M+NH_4_]^+^ | TG (53:4) | ●●● | ↑ |
|  | 896.7711 | [M+NH_4_]^+^ | TG (54:6) | ●●● | ↑ |
|  | 898.7863 | [M+NH_4_]^+^ | TG (54:5) | ●●● | ↑↑↑ |
|  | 900.8025 | [M+NH_4_]^+^ | TG (54:4) | ●●● | ↑↑ |
|  | 902.8184 | [M+NH_4_]^+^ | TG (54:3) | ●●● | ↑↑↑ |
|  | 920.7714 | [M+NH_4_]^+^ | TG (56:8) | ●● | ↑ |
|  | 924.8027 | [M+NH_4_]^+^ | TG (56:6) | ●● | ↑ |
|  | 928.8342 | [M+NH_4_]^+^ | TG (56:4) | ●● | ↑↑ |
|  | 948.8025 | [M+NH_4_]^+^ | TG (58:8) | ●●● | **↓↓** |
| **b. Glycerophospholipids** |  |  |  |  |  |
| Lyso-Phosphatidylcholine (LPC) | 468.3090 | [M+H]^+^ | LPC (14:0) | ● | ↑↑ |
|  | 518.3222 | [M+H]^+^ | LPC (18:3) | ●●● | **↓↓** |
|  | 520.3401 | [M+H]^+^ | LPC (18:2) | ●●● | **↓** |
|  | 544.3411 | [M+H]^+^ | LPC (20:4) | ●●● | ↑↑ |
| Phosphatidylcholine (PC) | 664.4913 | [M+H]^+^ | PC (27:0) | ●●● | **↓** |
|  | 720.5548 | [M+H]^+^ | PC (31:0) | ●●● | ↑ |
|  | 732.5503 | [M+H]^+^ | PC (32:1) | ● | **↓** |
|  | 734.5695 | [M+H]^+^ | PC (32:0) | ●●● | ↑ |
|  | 756.5546 | [M+H]^+^ | PC (34:3) | ●●● | ↑ |
|  | 758.5689 | [M+H]^+^ | PC (34:2) | ●●● | ↑↑↑ |
|  | 760.5840 | [M+H]^+^ | PC (34:1) | ●● | **↓↓** |
|  | 776.5237 | [M+H]^+^ | PC (36:7) | ●●● | ↑↑ |
|  | 778.5375 | [M+H]^+^ | PC (36:6) | ●●● | ↑↑ |
|  | 786.6010 | [M+H]^+^ | PC (36:2) | ●●● | **↓** |
|  | 814.6331 | [M+H]^+^ | PC (38:2) | ●●● | **↓** |
|  | 820.5865 | [M+H]^+^ | PC (39:6) | ●●● | **↓** |
|  | 836.6152 | [M+H]^+^ | PC (40:5) | ●●● | **↓** |
|  | 854.5703 | [M+H]^+^ | PC (42:10) | ●●● | **↓** |
|  | 856.5825 | [M+H]^+^ | PC (42:9) | ●● | **↓** |
|  | 860.6171 | [M+H]^+^ | PC (42:7) | ●●● | **↓↓** |
|  | 862.6329 | [M+H]^+^ | PC (42:6) | ●●● | **↓** |
|  | 870.6956 | [M+H]^+^ | PC (42:2) | ●●● | **↓↓↓** |
|  | 872.7115 | [M+H]^+^ | PC (42:1) | ●●● | **↓↓** |
|  | 884.6132 | [M+Na]^+^ | PC (42:6) | ●●● | **↓↓↓** |
|  | 898.7275 | [M+H]^+^ | PC (44:2) | ●●● | **↓↓** |
|  | 746.6063 | [M+H]^+^ | PC (O-34:1) | ●● | ↑↑ |
|  | 792.5940 | [M+H]^+^ | PC (O-38:6) | ●●● | **↓** |
| Phosphatidylethanolamine (PE) | 742.5380 | [M+H]^+^ | PE (36:3) | ●●● | ↑ |
|  | 764.5234 | [M+H]^+^ | PE (38:6) | ●●● | **↓** |
|  | 766.5383 | [M+H]^+^ | PE (38:5) | ●●● | **↓↓↓** |
|  | 788.5247 | [M+H]^+^ | PE (40:8) | ●●● | **↓** |
|  | 790.5391 | [M+H]^+^ | PE (40:7) | ●●● | **↓** |
| Phosphatidylserine (PS) | 750.5269 | [M+H]^+^ | PS (33:0) | ● | **↓** |
|  | 818.6256 | [M+H]^+^ | PS (P-39:0) | ●●● | ↑↑ |
|  | 848.5447 | [M+H]^+^ | PS (41:7) | ●●● | **↓** |
| **c. Sphingolipids** |  |  |  |  |  |
| Sphingomyelin (SM) | 813.6885 | [M+H]^+^ | SM (d18:1/24:1) | ●●● | **↓↓** |
|  | 829.7138 | [M+H]^+^ | SM (d18:1/25:0) | ●●● | **↓↓** |
|  | 841.7166 | [M+H]^+^ | SM (d18:1/26:1) | ●●● | **↓** |
| Ceramide (Cer) | 540.5359 | [M+H]^+^ | Cer (d18:0/16:0) | ●●● | ↑↑ |
|  | 562.5177 | [M+H]^+^ | Cer (d18:2/18:1) | ●●● | ↑↑ |
|  | 650.6455 | [M+H]^+^ | Cer (d18:0/24:1) | ●●● | ↑↑ |
|  | 668.6562 | [M+H]^+^ | Cer (d18:0/h24:0) | ●●● | **↓↓** |
|  | 680.6924 | [M+H]^+^ | Cer (d20:0/24:0) | ●●● | ↑↑ |
|  | 700.5731 | [M+H]^+^ | GlcCer (d18:1/16:0) | ●●● | **↓** |
|  | 814.7137 | [M+H]^+^ | GlcCer (d18:0/24:0) | ●●● | **↓** |
|  | 838.7137 | [M+H]^+^ | GlcCer (d18:1/26:1) | ●●● | **↓** |
|  | 840.7291 | [M+H]^+^ | GlcCer (d18:1/26:0) | ●●● | **↓↓** |
|  | 862.6287 | [M+H]^+^ | Galα1-4Galβ-Cer (d18:1/16:0) | ●● | **↓** |
|  |  |  |  |  |  |

^a^ Metabolites identification was matched with LIPIDMAPS based on its mass accuracy and fragmented spectral.

^b^ ●, *p*-value < 0.05; ●● *p*-value < 0.01; ●●● *p*-value < 0.001 determined by t-test (non-parametric analysis).

^c^ Fold changes of peak area relative to mean control. Log_2_ transformed. ↑, up-regulated ≥ 1 folds, ↑↑, up-regulated ≥ 2 folds; ↑↑↑, up-regulated ≥ 3 folds; ↑↑↑↑, up-regulated ≥ 4 folds; ↑↑↑↑↑, up-regulated ≥ 5 folds and *vice versa*.

**Supplementary Table S6. Significant changes of hydrophobic metabolites identified from SK-HEP-1 cells induced by 48 h treatment with *E. indica* root fraction (R-S5-C1-H1) using LC-MS/MS.**

| **Lipid Class** | **Detected m/z** | **Ion Adduct** | **Lipids Identification^a^** | ***p*-value^b^** | **Fold-changes^c^** |
| --- | --- | --- | --- | --- | --- |
| **a. Glycerolipids** |  |  |  |  |  |
| Diacylglycerol (DG) | 509.4179 | [M+H]^+^ | DG (28:2) | ●●● | ↑↑↑↑↑ |
|  | 551.5046 | [M+H]^+^ | DG (P-32:1) | ●●● | ↑↑↑ |
|  | 558.5101 | [M+NH_4_]^+^ | DG (30:0) | ●●● | **↓↓** |
|  | 589.5565 | [M+H]^+^ | DG (dO-36:4) | ●●● | ↑↑↑ |
|  | 603.5358 | [M+H]^+^ | DG (O-36:4) | ●●● | ↑↑↑↑ |
|  | 638.5728 | [M+NH_4_]^+^ | DG (36:2) | ●●● | ↑ |
|  | 640.5887 | [M+NH_4_]^+^ | DG (36:1) | ●●● | ↑ |
|  | 660.5573 | [M+NH_4_]^+^ | DG (38:5) | ●●● | **↓** |
|  | 662.5732 | [M+NH_4_]^+^ | DG (38:4) | ●●● | **↓↓** |
|  | 665.5129 | [M+H]^+^ | DG (40:8) | ●●● | ↑↑ |
|  | 689.5158 | [M+H]^+^ | DG (42:10) | ●●● | **↓↓↓↓↓** |
|  | 715.6207 | [M+H]^+^ | DG (43:4) | ●●● | ↑↑↑↑↑ |
| Triacylglycerols (TG) | 712.6467 | [M+NH_4_]^+^ | TG (40:0) | ●●● | **↓↓** |
|  | 752.6775 | [M+NH_4_]^+^ | TG (43:1) | ●●● | **↓↓↓** |
|  | 758.6261 | [M+NH_4_]^+^ | TG (44:5) | ●●● | **↓↓↓** |
|  | 768.7091 | [M+NH_4_]^+^ | TG (44:0) | ●●● | ↑ |
|  | 778.6918 | [M+NH_4_]^+^ | TG (45:2) | ●●● | ↑↑↑↑↑ |
|  | 818.7245 | [M+NH_4_]^+^ | TG (48:3) | ●●● | ↑ |
|  | 820.7405 | [M+NH_4_]^+^ | TG (48:2) | ●●● | ↑ |
|  | 834.7563 | [M+NH_4_]^+^ | TG (49:2) | ●●● | ↑ |
|  | 844.7405 | [M+NH_4_]^+^ | TG (50:4) | ●●● | ↑↑↑↑ |
|  | 846.7562 | [M+NH_4_]^+^ | TG (50:3) | ●●● | ↑↑ |
|  | 848.7719 | [M+NH_4_]^+^ | TG (50:2) | ● | ↑↑↑ |
|  | 860.7721 | [M+NH_4_]^+^ | TG (51:3) | ●●● | ↑↑ |
|  | 870.7516 | [M+NH_4_]^+^ | TG (52:5) | ●●● | ↑ |
|  | 872.7714 | [M+NH_4_]^+^ | TG (52:4) | ●●● | ↑↑↑↑ |
|  | 874.7876 | [M+NH_4_]^+^ | TG (52:3) | ●●● | ↑ |
|  | 886.7872 | [M+NH_4_]^+^ | TG (53:4) | ●●● | **↓** |
|  | 896.7706 | [M+NH_4_]^+^ | TG (54:6) | ●●● | ↑ |
|  | 898.7874 | [M+NH_4_]^+^ | TG (54:5) | ●●● | ↑↑↑ |
|  | 900.8030 | [M+NH_4_]^+^ | TG (54:4) | ●●● | ↑↑ |
|  | 914.8194 | [M+NH_4_]^+^ | TG (55:4) | ●●● | **↓↓↓** |
|  | 920.7723 | [M+NH_4_]^+^ | TG (56:8) | ●●● | ↑↑ |
|  | 924.8032 | [M+NH_4_]^+^ | TG (56:6) | ●●● | ↑ |
|  | 926.8189 | [M+NH_4_]^+^ | TG (56:5) | ●●● | ↑↑↑↑ |
|  | 928.8341 | [M+NH_4_]^+^ | TG (56:4) | ●●● | ↑ |
|  | 950.8188 | [M+NH_4_]^+^ | TG (58:7) | ●●● | ↑↑ |
| **b. Glycerophospholipids** |  |  |  |  |  |
| Lyso-Phosphatidylcholine (LPC) | 468.3091 | [M+H]^+^ | LPC (14:0) | ●●● | **↓** |
|  | 494.3248 | [M+H]^+^ | LPC (16:1) | ●●● | **↓** |
|  | 496.3409 | [M+H]^+^ | LPC (16:0) | ●●● | ↑ |
|  | 518.3225 | [M+H]^+^ | LPC (18:3) | ●●● | ↑↑ |
|  | 522.3563 | [M+H]^+^ | LPC (18:1) | ●●● | ↑ |
|  | 524.3718 | [M+H]^+^ | LPC (18:0) | ●●● | ↑ |
|  | 544.3393 | [M+H]^+^ | LPC (20:4) | ●● | **↓** |
|  | 480.3444 | [M+H]^+^ | LPC (O-16:1) | ●●● | **↓** |
| Phosphatidylcholine (PC) | 662.4767 | [M+H]^+^ | PC (27:1) | ●●● | ↑↑ |
|  | 664.4925 | [M+H]^+^ | PC (27:0) | ●●● | ↑↑↑ |
|  | 704.5240 | [M+H]^+^ | PC (30:1) | ●●● | ↑↑ |
|  | 706.5402 | [M+H]^+^ | PC (30:0) | ●●● | **↓↓↓** |
|  | 712.4930 | [M+H]^+^ | PC (31:4) | ●●● | **↓↓↓** |
|  | 714.5079 | [M+H]^+^ | PC (31:3) | ●●● | ↑↑↑ |
|  | 720.5554 | [M+H]^+^ | PC (31:0) | ●●● | ↑↑ |
|  | 728.5220 | [M+H]^+^ | PC (32:3) | ●●● | ↑↑↑ |
|  | 730.5394 | [M+H]^+^ | PC (32:2) | ●●● | ↑↑ |
|  | 734.5695 | [M+H]^+^ | PC (32:0) | ● | **↓↓** |
|  | 748.5869 | [M+H]^+^ | PC (33:0) | ●●● | ↑ |
|  | 756.5551 | [M+H]^+^ | PC (34:3) | ●●● | ↑↑↑ |
|  | 760.5834 | [M+H]^+^ | PC (34:1) | ●●● | **↓↓↓** |
|  | 762.6019 | [M+H]^+^ | PC (34:0) | ●●● | **↓↓↓** |
|  | 778.5396 | [M+H]^+^ | PC (36:6) | ●●● | ↑ |
|  | 782.5711 | [M+H]^+^ | PC (36:4) | ●● | **↓↓↓↓** |
|  | 786.6018 | [M+H]^+^ | PC (36:2) | ●●● | **↓↓↓↓** |
|  | 788.6084 | [M+H]^+^ | PC (36:1) | ●●● | **↓↓↓↓** |
|  | 806.5693 | [M+H]^+^ | PC (38:6) | ●●● | ↑ |
|  | 810.6006 | [M+H]^+^ | PC (38:4) | ●●● | **↓↓↓↓** |
|  | 820.5874 | [M+H]^+^ | PC (39:6) | ● | **↓↓↓** |
|  | 854.5742 | [M+H]^+^ | PC (42:10) | ●●● | **↓↓↓** |
|  | 864.6489 | [M+H]^+^ | PC (42:5) | ●●● | **↓↓↓** |
|  | 900.7435 | [M+H]^+^ | PC (44:1) | ●●● | **↓↓↓↓↓** |
|  | 714.5418 | [M+H]^+^ | PC (O-32:3) | ●●● | ↑ |
|  | 742.5734 | [M+H]^+^ | PC (O-34:3) | ●●● | ↑ |
|  | 746.6051 | [M+H]^+^ | PC (O-34:1) | ● | ↑↑ |
|  | 772.6232 | [M+H]^+^ | PC (O-36:2) | ●●● | **↓** |
|  | 796.6211 | [M+H]^+^ | PC (O-38:4) | ●●● | **↓** |
|  | 804.6860 | [M+H]^+^ | PC (O-38:0) | ● | **↓↓** |
|  | 738.5447 | [M+H]^+^ | PC (P-34:4) | ●●● | **↓↓↓↓** |
|  | 744.5917 | [M+H]^+^ | PC (P-34:1) | ●●● | **↓↓↓↓↓** |
|  | 794.6051 | [M+H]^+^ | PC (P-38:4) | ●●● | **↓** |
|  | 818.6069 | [M+H]^+^ | PC (P-40:6) | ●●● | **↓** |
| Phosphatidylethanolamine (PE) | 692.5238 | [M+H]^+^ | PE (32:0) | ●●● | ↑ |
|  | 742.5393 | [M+H]^+^ | PE (36:3) | ●●● | ↑ |
|  | 858.6968 | [M+H]^+^ | PE (44:1) | ●●● | **↓↓↓** |
|  | 622.4817 | [M+H]^+^ | PE (O-28:0) | ●●● | **↓↓** |
|  | 636.4966 | [M+H]^+^ | PE (O-29:0) | ●●● | **↓** |
|  | 648.4964 | [M+H]^+^ | PE (O-30:1) | ●●● | **↓↓↓** |
|  | 744.4960 | [M+H]^+^ | PE (O-38:9) | ●●● | **↓↓** |
|  | 724.5288 | [M+H]^+^ | PE (P-36:4) | ●●● | **↓↓↓↓↓** |
|  | 774.5477 | [M+H]^+^ | PE (P-40:7) | ●●● | **↓↓↓↓** |
| Phosphatidylserine (PS) | 774.5641 | [M+H]^+^ | PS (P-36:1) | ●●● | ↑↑↑↑↑ |
| **c. Sphingolipids** |  |  |  |  |  |
| Sphingomyelin (SM) | 677.5602 | [M+H]^+^ | SM (d18:0/14:0) | ●●● | ↑↑↑ |
|  | 703.5750 | [M+H]^+^ | SM (d16:1/18:0) | ●●● | **↓↓↓↓** |
|  | 789.6833 | [M+H]^+^ | SM (d18:0/22:0) | ●●● | ↑ |
|  | 811.6675 | [M+H]^+^ | SM (d18:2/24:1) | ●●● | **↓↓↓** |
| Ceramide (Cer) | 524.5410 | [M+H]^+^ | Cer (m18:0/16:0) | ●●● | **↓↓↓** |
|  | 540.5363 | [M+H]^+^ | Cer (d18:0/16:0) | ●●● | ↑↑↑↑↑ |
|  | 636.6303 | [M+H]^+^ | Cer (d18:1/23:0) | ●●● | **↓↓↓** |
|  | 650.6459 | [M+H]^+^ | Cer (d18:0/24:1) | ●●● | ↑↑↑ |
|  | 698.5581 | [M+H]^+^ | GlcCer (d18:2/16:0) | ●●● | **↓↓↓↓↓** |
|  | 700.5736 | [M+H]^+^ | GlcCer (d18:1/16:0) | ●●● | **↓↓** |
|  | 714.5888 | [M+H]^+^ | GlcCer (d15:1/20:0) | ●●● | **↓↓** |
|  | 716.5663 | [M+H]^+^ | GlcCer (d14:1/20:0(2OH)) | ● | **↓↓↓** |
|  | 728.6047 | [M+H]^+^ | GlcCer (d18:0/18:1) | ●●● | **↓↓** |
|  | 810.6833 | [M+H]^+^ | GlcCer (d18:1/24:1) | ●●● | **↓↓** |
|  | 812.6987 | [M+H]^+^ | GlcCer (d18:0/24:1) | ●●● | **↓↓↓** |
|  | 814.7132 | [M+H]^+^ | GlcCer (d18:0/24:0) | ●●● | ↑↑ |
|  | 862.6268 | [M+H]^+^ | Galα1-4Galβ-Cer (d18:1/16:0) | ●●● | ↑ |

^a^ Metabolites identification was matched with LIPIDMAPS based on its mass accuracy and fragmented spectral.

^b^ ●, *p*-value < 0.05; ●● *p*-value < 0.01; ●●● *p*-value < 0.001 determined by t-test (non-parametric analysis).

^c^ Fold changes of peak area relative to mean control. Log_2_ transformed. ↑, up-regulated ≥ 1 folds, ↑↑, up-regulated ≥ 2 folds; ↑↑↑, up-regulated ≥ 3 folds; ↑↑↑↑, up-regulated ≥ 4 folds; ↑↑↑↑↑, up-regulated ≥ 5 folds and *vice versa*.
